# Isolation, Structure Elucidation and Total Synthesis of Lajollamide A from the Marine Fungus *Asteromyces cruciatus*

**DOI:** 10.3390/md10122912

**Published:** 2012-12-19

**Authors:** Tobias A. M. Gulder, Hanna Hong, Jhonny Correa, Ekaterina Egereva, Jutta Wiese, Johannes F. Imhoff, Harald Gross

**Affiliations:** 1 Kekulé-Institute of Organic Chemistry and Biochemistry, University of Bonn, Gerhard-Domagk Str. 1, Bonn 53121, Germany; E-Mail: hhong@uni-bonn.de; 2 Institute for Pharmaceutical Biology, University of Bonn, Nussallee 6, Bonn 53115, Germany; E-Mails: jhonny.correa@utp.ac.pa (J.C.); eeguerev@uni-bonn.de (E.E.); 3 Kieler Wirkstoff-Zentrum (KiWiZ) at the Helmholtz-Zentrum für Ozeanforschung GEOMAR, Am Kiel-Kanal 44, Kiel 24106, Germany; E-Mails: jwiese@geomar.de (J.W.); jimhoff@geomar.de (J.F.I.); 4 Institute for Pharmaceutical Biology, University of Tübingen, Auf der Morgenstelle 8, Tübingen 72076, Germany

**Keywords:** fungus, *Asteromyces cruciatus*, cyclic peptides, OSMAC, total synthesis

## Abstract

The marine-derived filamentous fungus *Asteromyces cruciatus* 763, obtained off the coast of La Jolla, San Diego, USA, yielded the new pentapeptide lajollamide A (**1**), along with the known compounds regiolone (**2**), hyalodendrin (**3**), gliovictin (**4**), ^1^*N*-norgliovicitin (**5**), and bis-*N*-norgliovictin (**6**). The planar structure of lajollamide A (**1**) was determined by Nuclear Magnetic Resonance (NMR) spectroscopy in combination with mass spectrometry. The absolute configuration of lajollamide A (**1**) was unambiguously solved by total synthesis which provided three additional diastereomers of **1** and also revealed that an unexpected acid-mediated partial racemization (2:1) of the L-leucine and L-*N*-Me-leucine residues occurred during the chemical degradation process. The biological activities of the isolated metabolites, in particular their antimicrobial properties, were investigated in a series of assay systems.

## 1. Introduction

Filamentous fungi, particularly marine-derived fungi, are widely recognized as an emerging source for the production of novel and bioactive secondary metabolites [1,2,3,4]. This empirical fact is corroborated by the insights gained from recent fungal genome sequencing projects. These genomic studies attribute filamentous fungi an overall tremendous biosynthetic capacity because they commonly carry about 30 to 40 biosynthetic gene clusters coding for secondary metabolites [5,6]. However, the majority of these gene clusters have not yet been correlated to their corresponding natural products. Considering the given therapeutic value of fungal metabolites [1,2,3,4] and the huge amount of hitherto unexplored orphan and silent gene clusters, the activation and discovery of such biosynthetic loci represents a valuable strategy to obtain new bioactive products [7].

**Figure 1:**
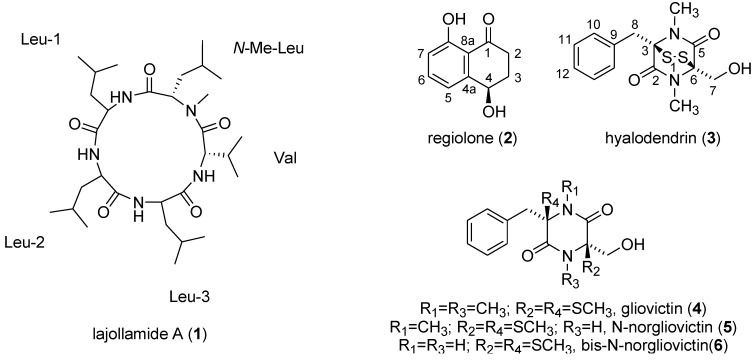
Secondary metabolites isolated from *Asteromyces cruciatus* 763.

Filamentous fungi of the genus *Asteromyces* comprise currently only one valid species and belong to the division of mitosporic fungi [8]. To date, aside from triglycerides and sterols [9,10], only the compounds gliovictin (**4**) [9,11], 2,4-dimethyl-4,5-dihydrofuran-3-carbaldehyde, 3*S*,5*R*-dihydrodimethylfuran-2-one, and cyclo-phenylalanine-serine [11] have been reported from different *Asteromyces cruciatus* samples. Fueled by the significantly high metabolic capacity of filamentous fungi and the fact that *Asteromyces* species are chemically underexplored, we performed a comprehensive chemical investigation with the strain *Asteromyces cruciatus* 763, obtained off La Jolla shore, San Diego, USA. In order to stimulate the production of secondary metabolites encoded by silent biosynthesis gene clusters and to enhance the production of constitutively produced secondary metabolites in our fungal isolate, we integrated the “One Strain Many Compounds” (OSMAC) approach [12,13,14,15] into our study. In this paper, we report the isolation of a new pentapeptide designated lajollamide A (**1**), and four known 3*R*,6*R* configured epipolythiopiperazinedione antibiotics hyalodendrin (**3**) [16], gliovictin (**4**) [9,11,16,17,18,19], ^1^*N*-norgliovictin (**5**) [18] and bis-*N*-norgliovictin (**6**) [18,19,20], in addition to the phytotoxic naphthalenone regiolone (**2**) [21,22] (See Figure 1). The elucidation of the absolute configuration of **1** has been a challenging problem and we report here the results of these efforts.

## 2. Results and Discussion

### 2.1. OSMAC Studies

A panel of extracts was prepared under 14 different culture conditions (different media composition, changing cultivation periods, extraction procedures, UV-light exposure, and temperature) and screened by LC-MS. Two whole broth extracts of *A. cruciatus* 763, that were grown either in a Czapek-Dox medium supplemented with cofactors or in a Czapek-Dox medium with an altered nitrogen source, afforded a good variety of known metabolites (e.g., cyclo-phenylalanine-serine and gliovictin [9,11]) and previously unknown compounds (e.g., *m/z* [M + H]^+^ = 566). The HPLC profiles of these two extracts indicated that they contained almost the same metabolites but with significant differences in their relative concentrations. Thus, the two cultivation conditions were separately scaled up and processed to isolate and characterize the major metabolites present in each of these extracts. 

### 2.2. Isolation and Structure Elucidation

The ethyl acetate extracts of the scaled-up fungal cultures were filtered through an RP-SPE cartridge and direct separation by RP-HPLC led to the isolation of compounds **1**–**6**. While the identity of the known metabolites **2**–**6** was established by direct comparison of the [α]_D_, MS, ^1^H and ^13^C NMR data with literature data, for the new compound **1** a complete structure determination study was performed.

**Figure 2:**
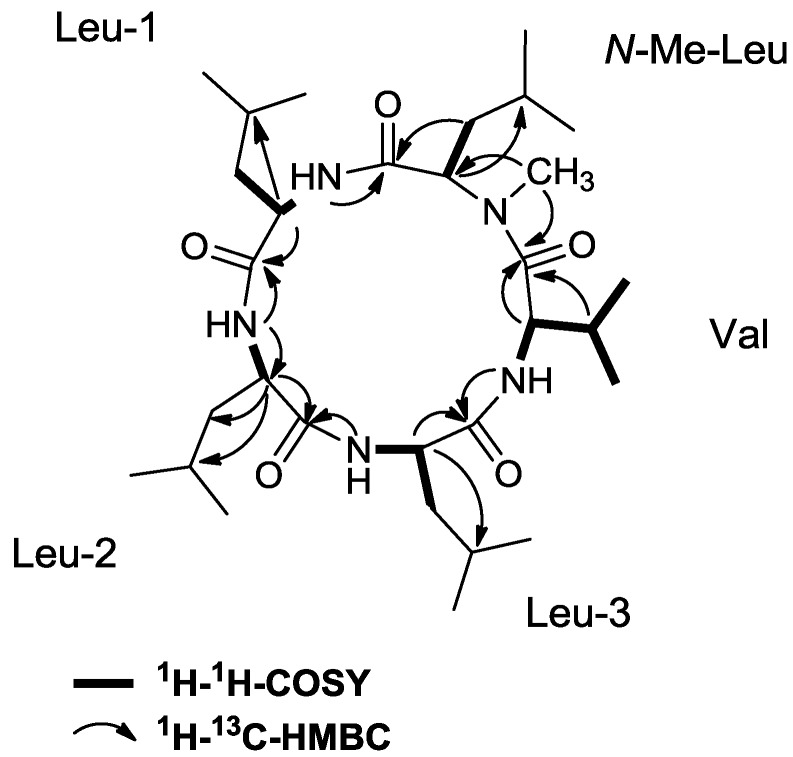
Key ^1^H–^1^H-COSY and ^1^H–^13^C-HMBC correlations leading to the identification of lajollamide A (**1**).

Compound **1** was assigned the molecular formula C_30_H_55_N_5_O_5_ via HR-ESI-MS data (*m/z* 566.4280 for [M + H]^+^), indicating a structure with six degrees of unsaturation. The IR spectrum showed absorption bands at 1633 cm^−1^, indicating the presence of amide functionalities. The ^1^H and ^13^C NMR spectra suggested that **1** was a peptide-type compound on the basis of chemical shifts and multiplicities typical for α-protons and carbons. In the ^1^H NMR spectrum, signals for five α-protons (δ_H_ 3.49, 4.23, 4.47, 4.48, and 4.58), 4 NH protons (δ_H_ 6.21, 6.92, 7.27, and 7.42) and an *N*-methyl group (δ_H_ 3.29) were observed. Most other resonances were found in the upfield region, including those for both methylene and methine protons (δ_H_ 1.37–2.33) and ten doublet methyl resonances (δ_H_ 0.85–1.02). The ^13^C and DEPT135 NMR spectra indicated 30 carbon resonances, consistent with the molecular formula derived from HR-ESI-MS, including five amide carbonyl carbons, ten methine carbons (five nitrogen-bearing), four methylene groups and eleven methyl groups (one nitrogen-bearing thereof) (Table 1). Interpretation of 1D and 2D NMR spectroscopic data of **1** allowed construction of five partial structures assigned as valine (Val), *N*-methylleucine (*N*-Me-Leu), and three leucine residues (Leu-1, Leu-2 and Leu-3). Resonances of each amino acid residue were identified by starting with an α carbon resonance in the region δ_C_ 50–65, locating the resonance of the α proton via the HSQC (Heteronuclear Single Quantum Coherence) spectrum, and using the COSY (Correlation Spectroscopy) spectrum to delineate the five independent spin systems formed by the respective amide and side chain protons of each amino acid. The resulting substructures were extended and corroborated by HMBC (Heteronuclear Multiple Bond Correlation) correlations (See Figure 2). 

**Table 1:** ^1^H and ^13^C NMR spectroscopic data for lajollamide A (**1**) ^a^.

Unit	Position	δ_C_^b^	δ_H_ (mult, *J* in Hz)
Leu-1	C=O	171.7, C	
	α	53.6, CH	4.23, m
	β	40.7, CH_2_	1.56, m; 1.77, m
	γ	24.9, CH	1.54 m
	δ	23.0, CH_3_ *^c^*	0.94, d ^c^
		21.3, CH_3_ *^c^*	0.90, d ^c^
	NH		6.21, brs
Leu-2	C=O	172.5, C	
	α	51.2, CH	4.48, m
	β	39.9, CH_2_	1.49, m; 1.86, m
	γ	25.2, CH	1.59, m
	δ	22.1, CH_3_^c^	0.86, d ^c^
		22.8, CH_3_^c^	0.90, d ^c^
	NH		7.42, d (8.3)
Leu-3	C=O	173.1, C	
	α	50.1, CH	4.58, m
	β	37.2, CH_2_	1.48, m; 1.69, m
	γ	24.6, CH	1.51, m
	δ	23.2, CH_3_^c^	0.92, d ^c^
		21.9, CH_3_^c^	0.88, d ^c^
	NH		7.27, m
Val	C=O	173.6, C	
	α	55.3, C	4.48, m
	β	30.3, CH	1.89, m
	γ	18.5, CH_3_	0.94
		19.2, CH_3_	0.92
	NH		6.92, d (8.9)
*N*-Me-Leu	C=O	171.2, C	
	α	65.2, CH	3.49, dd (4.1, 3.6)
	β	37.6, CH_2_	1.44, m; 2.24, m
	γ	25.3, CH	1.59, m
	δ	23.5, CH_3_^c^	0.96, d ^c^
		21.7, CH_3_^c^	0.97, d ^c^
	*N*-Me	40.9, CH_3_	3.29, s

^a^ Measured at 300 (^1^H) and 75 (^13^C) MHz in CDCl_3_; ^b^ Multiplicities were deduced from DEPT135 and HSQC experiments; ^c^ Assignments within a column may be interchanged.

The latter were key in the assignment of corresponding carbonyl groups, the γ-atoms of the leucine residues and the location of the *N*-methyl group. In this way, all resonances for each amino acid residue could be assigned apart from those for the Leu methyl groups, which could not be distinguished unambiguously from each other due to a heavy overlap of the methyl proton resonances. HMBC correlations from each NH/NCH_3_ resonance to one carbonyl carbon established the connectivity in the core of the structure (Figure 2): the NH signal of Leu-1 (δ_H_ 6.21) showed a long-range correlation to δ_C_ 171.2 which suggested that Leu-1 is connected to *N*-Me-Leu via its carbonyl group; further HMBC crosspeaks, this time between the signals of NH(Leu-2)/δ_C_ 171.7, NH(Leu-3)/δ_C_ 172.5, NH(Val)/δ_C_ 173.1 established the primary peptide sequence to be *N*-Me-Leu/Leu-1/Leu-2/Leu-3/Val. The ring closure was evident from ^1^H–^13^C couplings between the resonance of *N*-CH_3_ of *N*-Me-Leu and δ_C_ 173.6. These connectivities confirmed the cyclic pentapeptidic nature of **1**, thereby satisfying the required six elements of unsaturation. Based on the origin of the source microorganism and on its peptidic structure, **1** was given the name lajollamide A.

### 2.3. Initial Stereochemical Investigations

**Figure 3:**
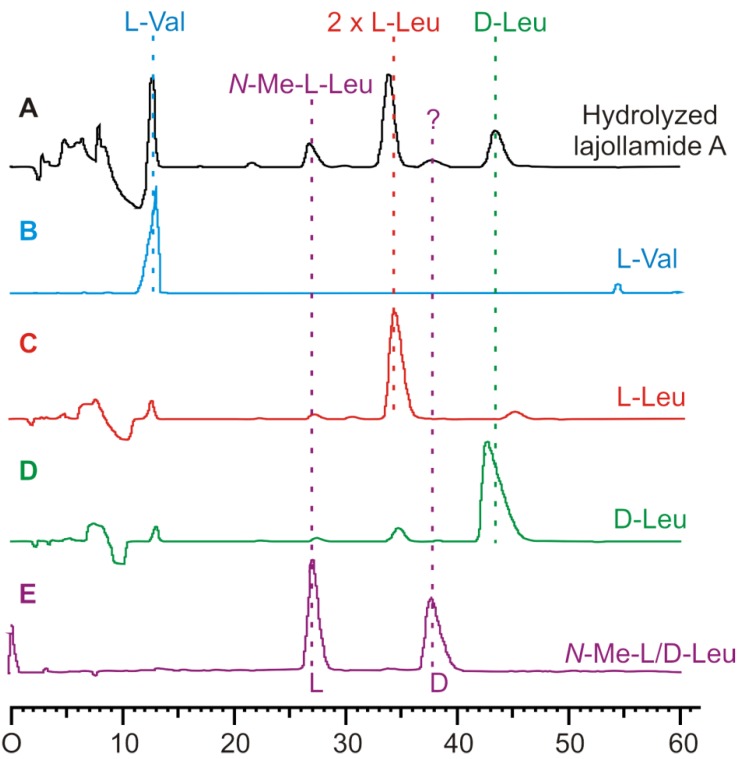
Stereochemical analysis of the amino acid building blocks in **1** by chiral HPLC after its acid-mediated hydrolysis. Selection of HPLC-UV traces used for assignments: (**A**) hydrolyzed lajollamide A (**1**, black), (**B**) L-Val (blue), (**C**) L-Leu (red), (**D**) D-Leu (green), and (**E**) *N*-Me-L/D-Leu (purple).

To elucidate the configurations at the stereogenic centers of the amino acid moieties in **1**, the natural product was fully hydrolyzed under acidic conditions. The hydrolysate obtained was analyzed by chiral HPLC. The stereochemical identity of the hydrolyzed amino acids observed in the HPLC chromatogram (Figure 3) was established by comparison with the respective commercial authentic standards of known absolute configuration. This led to the identification of *N*-Me-L-Leu, L-Val and three Leu residues, two of which are bearing L- and one D-configuration. The position of the expected D-Leu residue within the peptide backbone, however, was not evident from this, nor from the NMR spectroscopic data. To clarify this unsolved question and thus to fully stereochemically characterize **1**, we embarked on the total synthesis of the three possible diastereomers of this cyclic peptide.

### 2.4. Total Synthesis of Natural Lajollamide A (**1**) and of Its Diastereomeric Analogs B–D (**7–9**)

**Scheme 1:**
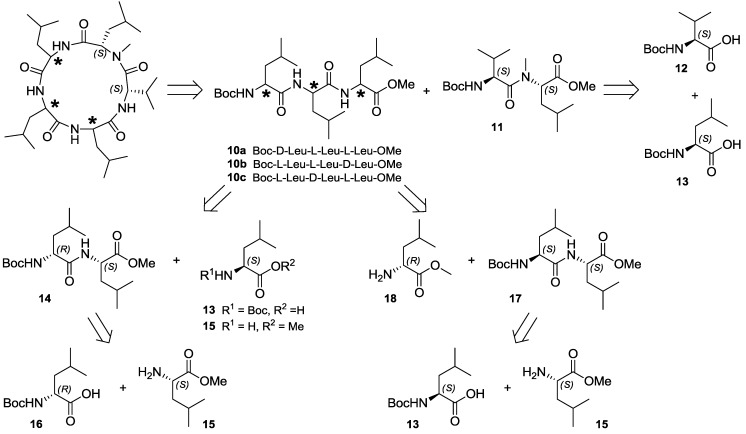
Retrosynthetic analysis for the preparation of all possible lajollamide diastereomers bearing one D-Leu unit.

In order to most efficiently access the three diastereomeric forms of lajollamide feasible based on the results of the peptide hydrolysis experiments, we chose a highly convergent synthetic approach applying solution phase peptide coupling chemistry. Retrosynthetically, the cyclic pentapeptide was divided into the three diastereomeric tripeptides **10a**–**c** and the stereochemically defined dipeptide building block **11** common to all target structures (Scheme 1). The latter can be derived of *N*-Boc-L-Val (**12**) and *N*-Boc-L-Leu (**13**). For the preparation of **10a** and **10c**, again a joint precursor dipeptide **14** could be defined, which is divergently transformable into the desired tripeptides by attaching *N*-Boc-L-Leu (**13**) or L-Leu-OMe (**15**) to its *N*- or *C*-terminus, respectively. The third fragment, **10b**, is accessible from D-Leu-OMe (**18**) and *N*-Boc-L-Leu-L-Leu-OMe (**17**). Dipeptides **14** and **17** are easily affordable from the commercially available amino acid precursors **13**, **15** and **16**.

**Scheme 2:**
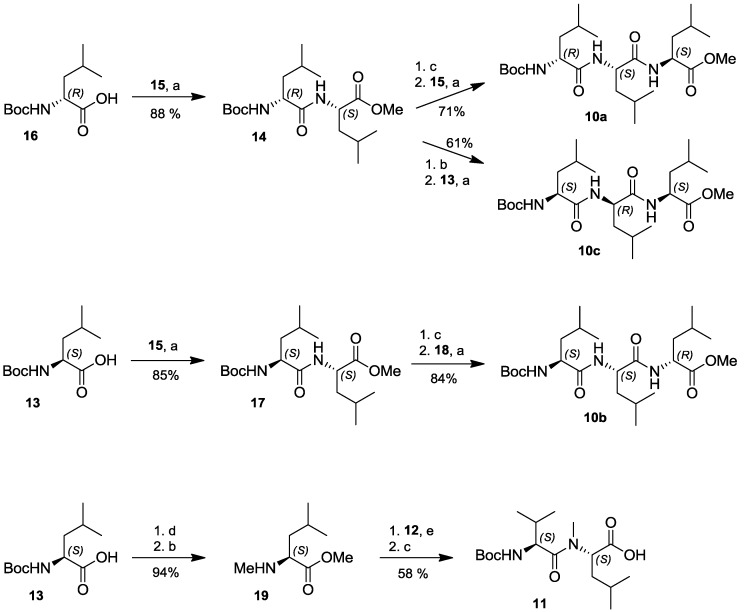
Synthesis of the tri- (**10a**–**c**) and dipeptide (**11**) building blocks of lajollamide A (**1**). a: HOBt, EDC, NEt_3_, CH_2_Cl_2_; b: AcCl, MeOH; c: 2N NaOH, MeOH; d: MeI, NaH, THF/DMF (10:1), 80 °C, 20h; e: HOBt, EDC, DIPEA, CH_2_Cl_2_.

The synthesis of the tri- (**10a**–**c**) and dipeptide (**11**) building blocks proceeded smoothly using standard peptide deprotection and coupling chemistry (Scheme 2): all peptide coupling steps were conducted using EDC and HOBt in CH_2_Cl_2_ with NEt_3_ as the base. Mild *N*-deprotection was achieved employing *in-situ* generation of HCl in MeOH by addition of acetylchloride. Saponification of the ester protective groups was generally carried out by applying 2 N NaOH in MeOH. Using these conditions, peptides **10a** and **10c** were prepared in three steps each, starting with **16** via dipeptide **14** in an overall yield of 62% and 54%, respectively. Analogously, **10b** was accessible from **13** in 71% overall yield. For the preparation of the universal dipeptide precursor **11**, *N*-Boc-L-Leu (**13**) was first dimethylated using MeI and NaH, with subsequent *N*-Boc removal to give **19**. This compound was coupled to **12** by using EDC and HOBt in CH_2_Cl_2_ with DIPEA as the base and saponified to furnish **11** in 55% overall yield.

**Scheme 3:**
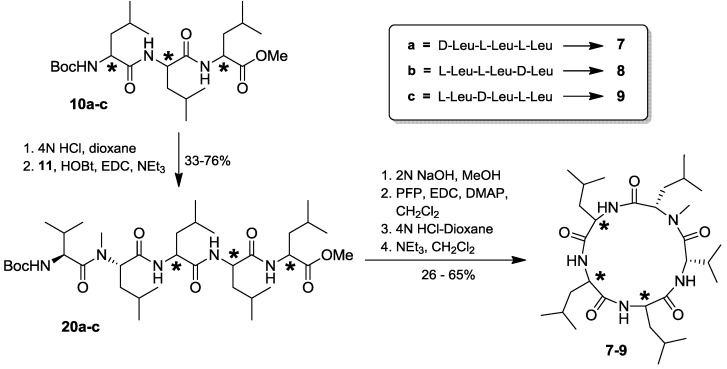
Assembly of the di- (**11**) and tripeptide (**10a**–**c**) building blocks to the target cyclopentapeptide structures **7**–**9**.

Having all desired building blocks in hands, we started the assembly of the three lajollamide diastereomers (Scheme 3). After removal of the *N*-Boc group in **10a**–**c** using 4N HCl in dioxane, coupling to **11** was again achieved with HOBt/EDC. The resulting fully protected pentapeptides **20a**–**c** were saponified, activated by PFP-ester formation and directly cyclized by *N*-deprotection followed by *in-situ* addition of NEt_3_, leading to compounds **7**–**9**.

**Figure 4:**
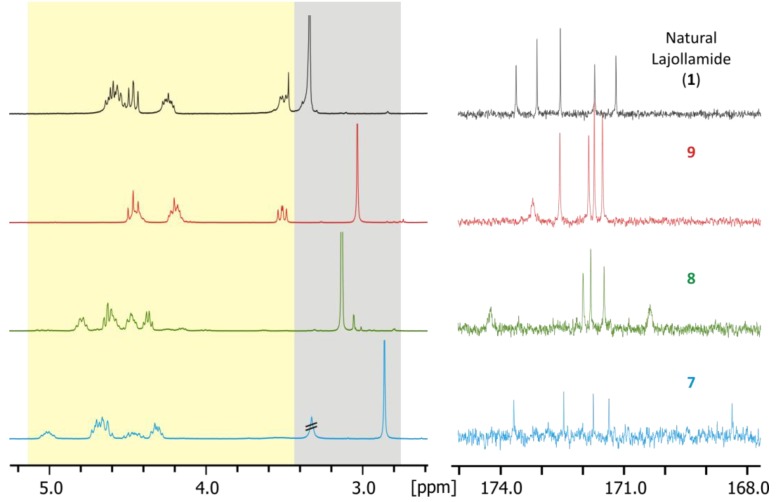
Comparison of selected signals in the ^1^H (left) and ^13^C (right) NMR spectra of natural lajollamide A (**1**, black), and compounds **7** (blue), **8** (green) and **9** (red). Shaded areas in the ^1^H spectra show α protons (yellow) and *N*-methyl groups (grey).

With the finalization of the synthesis of all anticipated lajollamide diastereomers **7**–**9**, we started to compare the spectroscopic data of the authentic natural product with that of the synthetic material. To our surprise, however, none of the peptides **7**–**9** was identical to the natural compound. This was clearly evident from substantial differences in chemical shifts in both ^1^H and ^13^C NMR spectra. For example, the chemical shifts of the amino acid α protons or the *N*-methyl groups differed significantly, as did all carbon chemical shifts, which was particularly apparent for the amide carbonyl signals (Figure 4).

**Figure 5:**
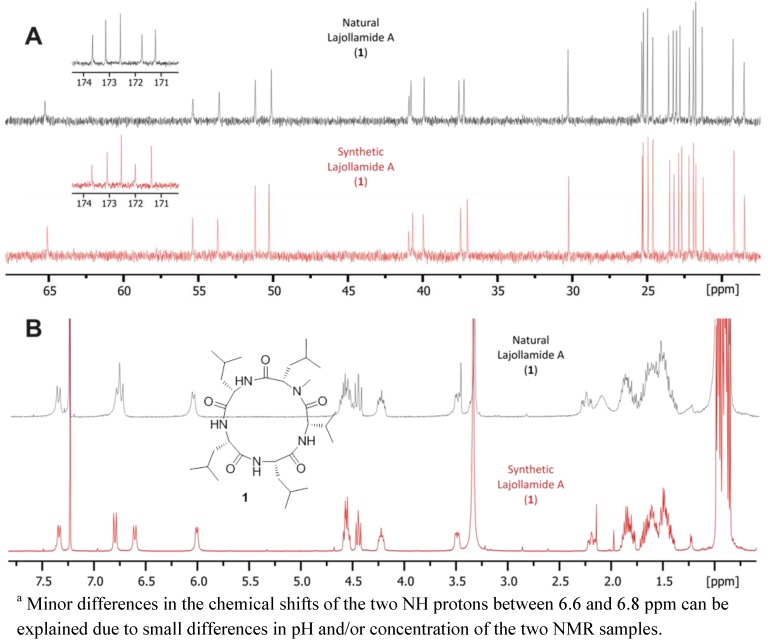
Comparison of the ^13^C (**A**) and ^1^H (**B**) NMR data of the isolated lajollamide A (**1**, black) with that of the identical, synthetically derived, all-L-configured material (red) ^a^.

This unexpected outcome of our synthetic endeavors led us to revisit the initial analytical data of the fungal metabolite. While no ambiguities were found in the interpretation of the NMR data, a small peak with a retention time of *ca.* 38 min. in the HPLC chromatogram of the full hydrolysate of the natural product drew our attention (see Figure 3). In fact, based on comparison with authentic standards, this signal turned out to correspond to *N*-Me-D-Leu. As the planar structure of lajollamide only contains one *N*-Me-Leu moiety, which was assigned to be L-configured due to the large peak at *ca*. 27 min in our stereochemical analysis, the occurrence of a fraction of *N*-Me-D-Leu clearly indicated a partial epimerization of this unit during peptide hydrolysis. This led to the assumption that a similar racemization process can also be expected for the three other Leu building blocks present in the molecule. This opened the possibility of three L-Leu units initially being present in the natural product, which, assuming an epimerization rate of approx. 30% for each of these residues, would result in a 2:1 ratio of L- to D-Leu in the analysis of the peptide hydrolysate, thus potentially explaining the spectroscopic differences of the natural product **1** when compared to the synthetic analogs **7**–**9**. With these thoughts in mind, the preparation of a lajollamide diastereomer bearing L-configuration at all amino acids residues was undertaken. The synthesis of the respective all L-tripeptide precursor **10d** followed the route depicted for the preparation of **10b** as shown in Scheme 2, altered only by simply attaching L-Leu-OMe to the *C*-terminus of **17** in the second peptide coupling step. Compound **10d** was obtained in overall 67% yield and further elaborated into the desired cyclic pentapeptide via all L-configured **20d** according to the sequence depicted in Scheme 3. Due to the convergent nature of our synthetic approach, the preparation of the all L-configured lajollamide-type target molecule was thus easily achieved with relatively little additional experimental work. To our great relief, this time, the analytical data for the synthetic material perfectly matched those of the natural product **1** (Figure 5). We thus unambiguously established the absolute configuration in natural **1**, henceforth named lajollamide A, to be all L. The three stereochmically altered, purely synthetic analogs of **1 **were consequently defined as lajollamides B–D (**7**–**9**).

### 2.5. Biological Activities of Compounds **1–9**

No cytotoxic or enzyme inhibitory activity was observed for compounds **1**–**9**. In antibacterial assays, metabolites **2**–**5** were found to be inactive. However compounds **1**, **7**, **8** and **9** exhibited a weak antibacterial activity against Gram-positive bacteria at a concentration of 100 μM. The growth inhibition of *Bacillus subtilis* was 61%, 51%, 67%, and 41%, respectively. Pathogenic bacterial strains causing infectious diseases were also inhibited. Inhibition of *Staphylococcus epidermidis* was shown for compound **1** (30%), **7** (43%) and **8** (32%). Only compound **7** was active against methicillin-resistant *Staphylococcus aureus* (MRSA). Furthermore, in agar diffusion assays at the 50 μg level, potent antibacterial and antifungal properties towards *Escherichia coli* (2.5 mm total inhibition), *Bacillus megaterium* (7 mm total inhibition), *Mycotypha microspora* (13.5 mm total inhibition), *Eurotium rubrum* (4 mm total inhibition), and *Microbotryum violaceum* (13 mm total inhibition) were demonstrated by **6**, which in some cases surpassed the activity of the positive controls streptomycin, benzylpenicillin and miconazole. 

## 3. Experimental Section

### 3.1. General Experimental Procedures

Optical rotations were measured on a Jasco DIP 140 polarimeter. UV and IR spectra were obtained using Perkin-Elmer Lambda and Perkin-Elmer spectrum BX instruments, respectively. All NMR spectra of the isolated material were recorded on a Bruker Avance 300 DPX spectrometer. Spectra of the synthetic compounds were acquired on Bruker AM 300 and AM 400 spectrometers. Spectra were referenced to residual protonated solvent signals with resonances at δ_H/C_ 3.35/49.0 (CD_3_OD) or 7.26/77.0 (CDCl_3_), respectively. HR-EI and HR-ESI-MS data was recorded on Kratos MS50, a Bruker Daltonics micrOTOF-Q and a Thermo Finnigan MAT 95 XL. Semipreparative HPLC was carried out using a Waters system consisting of a degasser, a 600 pump, a 996 photodiode array detector, and a 717 plus autosampler in combination with a Waters fraction collector. Chiral HPLC was performed on a Merck-Hitachi system consisting of a L-6200 A pump, a L-4500 A photodiode array detector (PDA), and a D-6000 A interface. LC-MS measurements were obtained by employing an Applied Biosystems LC/MS system consisting of an Agilent 1100 HPLC system and an MDS Sciex API 2000 mass spectrometer equipped with an API-ESI source. All solvents used were distilled under argon and dried over 3 Å molecular sieves prior to use. All amino acids, 1-(3-dimethylaminopropyl)-3-ethylcarbodiimide (EDC), pentafluorophenol (PFP) were purchased from Carbolution Chemicals. 1-Hydroxybenzotriazole hydrate (HOBt∙H_2_O) was purchased from Aldrich, triethylamine (NEt_3_) from Grüssing GmbH and methyl iodide from Merck. Sodium hydride and 4N HCl/dioxane were delivered from Acros Organics. All chemicals were used without further purification. For thin layer chromatography TLC Silica gel 60 F254 from Merck was used. The dyeing reagent consisted of ninhydrin in ethanol. For column chromatography, silica gel was purchased from Merck.

### 3.2. Biological Material

A piece of an unidentified decaying green alga, floating in the water column at a depth of 20 feet was collected by scuba in 2005 at La Jolla shore, San Diego, USA. Following transport in sterile seawater to Germany, the fungal strain #763 was isolated among other fungi from the algal sample, freed from competing microorganisms and other contaminants as previously described [23,24,25] and deposited at the fungal culture collection of the Institute for Pharmaceutical Biology, University of Bonn, Germany. The fungus was identified as *Asteromyces cruciatus* Moreau *et* Moreau *ex* Hennebert 1962. 

### 3.3. Screening of Secondary Metabolites Biosynthesized under Different Conditions (OSMAC Aproach)

The OSMAC approach was based on cultures of the fungus in media derived from the basic components of the Czapek-Dox broth [26,27]: NaNO_3_ (3 g/L), FeSO_4_ (0.01 g/L), KCl (0.5 g/L), MgSO_4_ (0.5 g/mL), K_2_HPO_4_ (1.0 g/L), carbon source (30 g/L, originally sucrose, however, when not indicated, glucose was used) in artificial sea water (ASW). For the preparation of the culture medium, all components except the carbon source were mixed in 250 mL (1 L capacity, baffled Erlenmeyer flasks) of ASW and the mixtures were autoclaved (121 °C, 20 min). Subsequently, 20 mL of 40% (m/V) carbon source were added by sterile filtration to the medium, followed by inoculation with small pieces of seed culture. Small agar pieces (1 × 1 cm) of a 12–25-day-old culture of *A. cruciatus* grown on biomalt salt agar medium served as seed cultures. In order to find culture conditions conducive to production of new metabolites, two replicate cultures of *A. cruciatus* were grown in each of 14 different culture conditions: (#1) Czapek-Dox medium with shaking (#2) Czapek-Dox medium without shaking for generation of low O_2_ levels [28], (#3) dixenic cultivation [29] with 10 mL of the bacterium *Pseudomonas fluorescens* Pf-5 [30], added on day 6, (#4) Czapek-Dox medium containing 20 g/L Amberlite XAD-16, (#5) Czapek-Dox medium supplemented with 1 mL/L cofactor solution consisting of MnCl_2_·4H_2_O (100 mg), CoCl_2_ (20 mg), CuSO_4_ (10 mg), Na_2_MoO_4_·2H_2_O (10 mg), ZnCl_2_ (20 mg), LiCl (5 mg), SnCl_2_·2H_2_O (5 mg), H_3_BO_3_ (10 mg), K_3_BO_3_ (10 mg), KBr (20 mg), KI (20 mg), EDTA-Na^+^ (5.8 mg) in 1 L of distilled water [28], (#6) Czapek-Dox supplemented with 4.6 mg/L CdCl_2_ as polyketide inducer [31], (#7) Czapek-Dox medium, cultivated from day 10 to 13 under elevated UV-B light exposition, (#8) Czapek-Dox medium, cultivated from day 8–10 at 35 °C [28], (#9) Czapek-Dox medium, cultivated with sub-inhibitory concentrations of 0.4 mL/L cycloheximide [32,33], (#10) Czapek-Dox medium, supplemented with 8 mL/L toluene, (#11) Czapek-Dox without NaNO_3_, containing 1.0 g/L Arg, 2.5 g/L Asn and 1.5 g/L Glu [28], (#12) Czapek-Dox with reduced levels of K_2_HPO_4_ (228.2 mg/L) for phosphate repression [28], (#13) Czapek-Dox medium without FeSO_4_ for induction of siderophores, (#14) Czapek-Dox medium with sucrose instead of glucose as carbon source [28]. If not stated otherwise, the cultures were grown with shaking (120 rpm) at 24 °C. The broth was harvested at 10 days after inoculation. The culture broths of each fermentation were extracted separately with EtOAc (2 × 250 mL), and evaporated under reduced pressure to afford dark brown semisolids, ranging from 0.3 to 47.2 mg. In the case of culture condition #4, additionally the medium polarity compounds absorbed by the XAD resin material were eluted by shaking, first for 1 h in 200 mL EtOAc, and subsequently for 1 h in 200 mL acetone. All resulting three organic crude extracts of cultivation experiment #4 were combined. The resultant crude extracts of each experiment were dissolved in methanol to a final concentration of 5 mg/mL, and 10 μL was profiled by LC/MS using a 2 mM NH_4_OAc buffered MeOH/H_2_O gradient, increasing the MeOH portion from 10% to 100% over 20 min and holding it at 100% MeOH for additional 10 min (Macherey-Nagel C_18_ Nucleodur 100-5 column, 2 × 125 mm, 5 μm column; 0.25 mL/min flow rate, with total ion current and photodiode array monitoring at 200–400 nm). 

### 3.4. Scaled-Up Cultivation, Extraction and Isolation of Metabolites of *A. cruciatus*

The fungus was cultivated for ten days in five 5 L-Erlenmeyer flasks, each containing 1.5 L of liquid media (=7.5 L). The liquid medium consisted of Czapek-Dox medium with glucose (30 g/L) as carbon source which was either supplemented with co-factors or contained solely the amino acids Arg, Asn and Glu as nitrogen source instead of NaNO_3_. Flasks were shaken on a rotary shaker incubator at 24 °C and 120 rpm. On day 10, the cultivation medium and mycelium of each experiment was extracted with EtOAc (2 × 7.5 L). After evaporation of the organic solvent, both crude extracts (medium with trace elements: 126 mg; medium with an altered nitrogen source: 98 mg) were re-suspended separately in MeOH and filtered through reversed phase silica gel (RP-SPE cartridge, 100 mg). Separation of each filtrate by RP-HPLC analysis using a linear gradient of 40:60–100:0 MeOH-H_2_O over a period of 54 min (column: Knauer Eurospher-100-C_18_; 8 × 250 mm, 5 μm; 2 mL/min; DAD detection) yielded collectively a compound (amount from trace element medium/amount from altered nitrogen source) **1** (18.6/7.1 mg), **2** (0.9/0.7 mg), **3** (1.6/3.3 mg), **4** (19.8/19.4 mg), **5** (1.8/0.9 mg) and **6** (1.0/3.2 mg). 

lajollamide A (**1**): yellowish glass; [α]_D_^24^ −80 (*c* 0.17, MeOH); UV/VIS (MeOH) λ_max_ (ε): 204 (12,600); IR (ATR): 3284, 2956, 1633, 1530 cm^−1^; HR-ESI-MS *m/z* 566.4280 [M + H]^+^ (calc. for C_30_H_56_N_5_O_5_, 566.4276, Δ +0.7 ppm); ^1^H and ^13^C NMR data in CDCl_3_, see Table 1. 

regiolone, syn. (−)-isosclerone (**2**): Colorless film; [α]_ D_^25^ −16 (*c* 0.02, MeOH), {lit. [21] [α]_D_ −68 (*c* 0.05, MeOH)}; UV/VIS (MeOH) λ_max_ (ε): 212 (15,800), 262 (9900), 324 (4200); ^1^H NMR (300 MHz, CDCl_3_) δ_H_ ppm: 1.87 (1H, brs, OH-4), 2.19 (1H, m, H-3a), 2.34 (1H, m, H-3b), 2.65 (1H, m, H-2a), 3.01 (1H, m, H-2b), 4.92 (1H, m, H-4), 6.93 (1H, d, 8.5 Hz, H-7), 7.02 (1H, d, 7.4 Hz, H-5), 7.50 (1H, dd, 7.4, 8.5 Hz, H-6), 12.43 (1H, s, OH-8); ^13^C NMR (75 MHz, CDCl_3_) δ_C_ ppm: 31.2 (CH_2_, C-3), 34.6 (CH_2_, C-2), 67.7 (CH, C-4), 115.2 (qC, C-8a), 117.3 (CH, C-5), 117.8 (CH, C-7), 137.0 (CH, C-6), 145.8 (qC, C-4a), 162.7 (qC, C-8), 204.2 (qC, C-1); LR-ESI-MS *m/z* 177.1 [M − H]^−^.

(3*R*,6*R*)-hyalodendrin, syn. A26771A (**3**): Yellowish film; [α]_ D_^24^ −71 (*c* 0.15, CHCl_3_) {lit. [16] [α]_D_^27^ −88 (*c* 0.15, MeOH)}; ^1^H NMR (300 MHz, CDCl_3_) δ_H_ ppm: 2.97 (3H, s, ^4^NCH_3_), 3.21 (3H, s, ^1^NCH_3_), 3.61 (1H, d, 16 Hz, H-8a), 4.08 (1H, d, 16 Hz, H-8b), 4.35 (2H, m, H_2_-7), 7.30 (5H, m, H-10, H-11, H-12, H-13, H-14); ^13^C NMR (75 MHz, CDCl_3_) δ_C_ ppm: 27.5 (^4^NCH_3_), 28.6 (^1^NCH_3_), 36.8 (CH_2_, C-8), 61.2 (CH_2_, C-7), 75.2 (qC, C-6), 75.7 (qC, C-3), 127.4 (CH, C-12), 128.7 (2 × CH, H-10, H-14), 129.0 (2 × CH, H-11, H-13), 134.0 (qC, C-9), 165.5 (qC, C-2), 166.9 (qC, C-5); HR-ESI-MS *m/z* 347.0491 [M + Na]^+^ (calc. for C_14_H_16_N_2_NaO_3_S_2_, 347.0495, Δ −1.2 ppm).

(3*R*,6*R*)-gliovictin, syn. A26771E, (3*R*,6*R*)-bisdethiodi(methylthio)hyalodendrin (**4**): Colorless film; [α]_D_^24^ −58 (*c* 0.88, CHCl_3_) {lit. [17] [α]_D_^25^ −65 (CHCl_3_); [9] [α]_D_ −62 (*c* 1.88, CHCl_3_); [16] [α]_D_^27^ −47 (*c* 0.13, CH_3_OH); [18] [α]_D_^24^ −43 (*c* 0.24, CHCl_3_)}; ^1^H NMR (300 MHz, CDCl_3_) δ_H_ ppm: 1.24 (1H, t, 6.9 Hz, OH-7), 2.13 (3H, s, ^6^SCH_3_), 2.30 (3H, s, ^3^SCH_3_), 3.02 (3H, s, ^4^NCH_3_), 3.10 (1H, dd, 6.9 and 11.8 Hz, H-7a), 3.13 (1H, d, 13.8 Hz, H-8a), 3.31 (3H, s, ^1^NCH_3_), 3.71 (1H, d, 13.8 Hz, H-8b), 3.85 (1H, dd, 6.9 and 11.8 Hz, H-7b), 7.09–7.27 (5H, m, H-10, H-11, H-12, H-13, H-14). ^13^C NMR (75 MHz, CDCl_3_) δ_C_ ppm: 13.4 (^6^SCH_3_), 14.3 (^3^SCH_3_), 29.3 (^1^NCH_3_), 31.0 (^4^NCH_3_), 42.3 (CH_2_,C-8), 64.3 (CH_2_, C-7), 71.5 (qC, C-6), 73.7 (qC, C-3), 127.9 (CH-12), 128.7 (2 × CH, C-11 and C-13), 130.1 (2 × CH, C-10 and C-14), 134.1 (qC, C-9), 165.3 (qC, C-2), 165.5 (qC, C-5); HR-ESI-MS *m/z* 377.0965 [M + Na]^+^ (calc. for C_16_H_22_N_2_NaO_3_S_2_, 377.0970, Δ −1.3 ppm).

(3*R*,6*R*)-^1^*N*-norgliovictin, syn. bisdethiodi(methylthio)-1-demethylhyalodendrin (**5**): Colorless film; [α]_D_^24^ −37 (*c* 0.05, CH_3_OH) {lit. [18] [α]_ D_^24^ −63 (*c* 0.30, CHCl_3_)}; ^1^H NMR (300 MHz, CDCl_3_) δ_H_ ppm: 1.71 (1H, brs, OH-7); 2.19 (3H, s, ^3^SCH_3_), 2.22 (3H, s, ^6^SCH_3_), 2.71 (1H, d, 11.6 Hz, H-7a), 3.15 (1H, d, 13.8 Hz, H-8a), 3.28 (3H, s, ^4^NCH_3_) , 3.41 (1H, d, 11.6 Hz, H-7b), 3.53 (1H, d, 13.8 Hz, H-8b), 6.36 (1H, brs, NH-1), 7.09–7.32 (5H, m, H-10, H-11, H-12, H-13, H-14); ^13^C NMR (75 MHz, CDCl_3_) δ_C_ ppm: 13.5 (^6^SCH_3_), 14.4 (^3^SCH_3_), 30.3 (^4^NCH_3_), 42.3 (CH_2_, C-8), 64.8 (qC, C-6), 65.2 (CH_2_, C-7), 75.7 (qC, C-3), 128.0 (CH, C-12), 128.8 (2 × CH, C-10, C-14), 130.0 (2 × CH, C-11, C-13), 133.7 (qC, C-9), 164.9 (2 × qC, C-2, C-5); HR-ESI-MS *m/z* 363.0818 [M + Na]^+^ (calc. for C_15_H_20_N_2_NaO_3_S_2_, 363.0813, Δ +1.4 ppm).

(3*R*,6*R*)-bis-*N*-norgliovictin (**6**): [α]_ D_^24^ −8 (*c* 0.1, CH_3_OH), {lit. [20] [α]_D_ −32 (*c* 0.1, CH_3_OH); [18] [α]_D_^25^ −34 (*c* 0.30, dioxane)}; ^1^H NMR (300 MHz, CH_3_OD) δ_H_ ppm: 2.23 (3H, s, ^6^SCH_3_), 2.37 (3H, s, ^3^SCH_3_), 3.05 (H, d, 11.5 Hz, H-7a), 3.07 (1H, d, 13.3 Hz, H-8a), 3.40 (H, d, 11.5 Hz, H-7b), 3.62 (1H, d, 13.3 Hz, H-8b), 7.29 (5H, m, H-10, H-11, H-12, H-13, H-14); ^13^C NMR (75 MHz, CH_3_OD) δ_C_ ppm: 13.5 (^3^SCH_3_ or ^6^SCH_3_), 13.9 (^3^SCH_3_ or ^6^SCH_3_), 46.0 (CH_2_, C-8), 66.5 (CH_2_, C-7), 67.8 (qC, C-3 or C-6), 68.8 (qC, C-3 or C-6), 128.4 (CH, C-12), 129.4 (2 × CH, C-11, C-13), 131.9 (2 × CH, C-10, C-14), 135.7 (qC, C-9), 167.8 (qC, C2 or C5), 168.2 (qC, C-2 or C-5); HR-ESI-MS *m/z* 349.0651 [M + Na]^+^ (calc. for C_14_H_18_N_2_NaO_3_S_2_, 349.0657, Δ −1.7 ppm).

### 3.5. Amino Acid Analysis by Chiral HPLC

Lajollamide A (1.3 mg) was hydrolyzed with 6 N HCl in a sealed vial at 108 °C for 19 h. Excess aqueous HCl was removed under vacuum and the resulting hydrolysate was analyzed by chiral HPLC employing a Phenomenex Chirex 3126 (D) column (4.6 × 250 mm) eluting with 2 mM CuSO_4_/MeCN (95:5) at a flow of 0.8 mL/min, monitoring at 240 nm. Comparison with commercially available standards suggested the presence of L-Val, *N*-Me-L-Leu, 2 × L-Leu and 1 × D-Leu. The hydrolysate was chromatographed alone and co-injected with standards to confirm assignments.

### 3.6. General Synthetic Protocols for the Preparation of Lajollamides A–D (**1, 7–9**)

#### 3.6.1. Peptide Coupling (Protocol A)

The amine (1 equivalent) was dissolved in CH_2_Cl_2_. After cooling to 0 °C, the acid (1.1 equivalent), HOBt (1 equivalent), EDC (1.25 equivalent) and NEt_3_ (5 equivalent) were added successively to the solution. The reaction mixture was stirred at room temperature overnight. The solvent was removed under vacuum. The crude product was taken up in water and extracted with CH_2_Cl_2_. The organic layers were combined and washed successively with saturated aqueous NaHCO_3_ solution, 40% citric acid solution, brine and dried over MgSO_4_. The solvent was removed under reduced pressure and the crude product purified by column chromatography.

#### 3.6.2. *N*-Deprotection (Protocol B)

##### 3.6.2.1. Deprotection with Acetyl Chloride in Methanol

*N*-Boc-protected peptide ester (1 equivalent) was dissolved in MeOH (24 equivalent). After cooling to 0 °C, acetyl chloride (2.2 equivalent) was added dropwise to the reaction solution which was further stirred at room temperature until completion of *N*-Boc removal as indicated by TLC. After solvent evaporation the crude product was taken up in CH_2_Cl_2_ and neutralized with saturated NaHCO_3_ solution. The aqueous layer was extracted with CH_2_Cl_2_. The organic layers were combined and dried over anhydrous MgSO_4_. After filtration the solvent was removed under high vacuum. 

##### 3.6.2.2. Deprotection with 4 M HCl∙Dioxane

*N*-Boc-protected peptide ester (1 equivalent) was treated with 4 M HCl/dioxane (100 equivalent) under argon and stirred at room temperature for 1 h. The resulting reaction mixture was concentrated under high vacuum.

#### 3.6.3. Saponification (Protocol C)

*N*-Boc-protected peptide ester (1 equivalent) was dissolved in MeOH (60 equivalent) und treated with 2 N NaOH (65 equivalent) solution. The reaction mixture was stirred at room temperature until the saponification finished as indicated by TLC. After solvent evaporation the crude product was taken up in CH_2_Cl_2_ and washed with H_2_O. The aqueous phase was acidified with 1 N HCl to pH 2 and extracted with CH_2_Cl_2_. The combined organic layers were dried over anhydrous MgSO_4_ and the solvent was removed under high vacuum. 

#### 3.6.4. Macrolactonization (Protocol D)

A fully protected pentapeptide (1 equivalent) was treated with 2 N NaOH following general protocol c. The resulting product was dissolved in CH_2_Cl_2_. After cooling to −10 °C EDC (1.3 equivalent), PFP (3 equivalent) and DMAP (0.1 equivalent) were added. The reaction mixture was stirred at room temperature overnight. After solvent evaporation and purification by column chromatography the resulting activated PFP ester was treated with 4 N HCl/dioxane (100 equivalent) following general protocol b2 to give the precursor for macrocyclisation. A highly dilute solution of the PFP ester (1 equivalent) in CH_2_Cl_2_ (0.003–0.01 M) was added dropwise to a solution of NEt_3_ (20 equivalent) in CH_2_Cl_2_. The resulting solution was stirred at room temperature overnight. After solvent evaporation the crude product was taken up in H_2_O and extracted with CH_2_Cl_2_. The combined organic layers were washed with 40% citric acid solution and brine and dried over anhydrous MgSO_4_. After filtration the solvent was removed under high vacuum. 

### 3.7. Total Synthesis of Lajollamides A–D (**1, 7–9**)

#### 3.7.1. *N*-Boc-L-Leu-L-Leu-OMe (**17**)

Peptide coupling of 10.0 g (40.1 mmol) *N*-Boc-L-Leu (**13**) with 6.4 g (44.1 mmol) L-Leu-OMe (**15**) was conducted following general protocol a, yielding 12.2 g (34.1 mmol, 85%) of the desired product **17** after chromatography on silica gel using ethylacetate and cyclohexane in 1:1 ratio as the eluent. ^1^H NMR (400 MHz, CDCl_3_) δ_H_ ppm: 0.85–0.98 (12H, m), 1.42 (9H, s), 1.51–1.65 (6H, m), 3.71 (3H, s), 4.09 (1H, brs), 4.59 (1H, m), 4.92 (1H, brs), 6.49 (1H, d, 6.5 Hz); ^13^C NMR (100 MHz, CDCl_3_) δ_C_ ppm: 21.8, 22.8, 22.8, 24.6, 24.6, 24.7, 28.2, 40.8, 41.5, 50.6, 52.2, 52.9, 80.2, 155.6, 172.2, 173.1; ESI-MS *m/z* 381.2 [M + Na]^+^, 281.2 [M + Na − C_5_H_9_NO_2_]^+^.

#### 3.7.2. *N*-Boc-D-Leu-L-Leu-OMe (**14**)

Peptide coupling of 4.99 g (20.0 mmol) *N*-Boc-D-Leu (**16**) with 3.2 g (22.0 mmol) L-Leu-OMe (**15**) was conducted following general protocol a, yielding 6.3 g (17.6 mmol, 88%) of the desired product **14** after chromatography on silica gel using ethyl acetate and cylcohexane in 1:1 ratio as the eluent. ^1^H NMR (400 MHz, CDCl_3_) δ_H_ ppm: 0.87–0.95 (12H, m), 1.43 (9H, s), 1.51–1.65 (6H, m), 3.70 (3H, s), 4.13 (1H, m), 4.57 (1H, m), 4.94 (1H, brs), 6.67 (1H, brs); ^13^C NMR (100 MHz, CDCl_3_) δ_C_ ppm: 21.9, 22.9, 23.0, 24.8, 24.9, 24.9, 28.4, 41.1, 41.5, 50.8, 52.4, 53.1, 80.2, 155.8, 172.6, 173.4; ESI-MS *m/z* 381.2 [M + Na]^+^, 281.2 [M + Na − C_5_H_10_N]^+^.

#### 3.7.3. *N*-Boc-D-Leu-L-Leu-L-Leu-OMe (**10a**)

6 g (16.7 mmol) *N*-Boc-D-Leu-L-Leu-OMe (**14**) were saponified following general protocol c. The success of the deprotection step was monitored by ^1^H NMR of the resulting crude product (^1^H data for *N*-Boc-D-Leu-L-Leu: (400 MHz, CDCl_3_) δ_H_ ppm: 0.88–0.97 (12H, m), 1.41 (9H, s), 1.57–1.67 (6H, m), 4.41 (1H, m), 4.60 (1H, m), 5.39 (1H, m), 7.11 (1H, brs), 10.55 (1H, brs)). This material was used in the peptide coupling step according to general protocol a with 2.1 g (14.8 mmol) L-Leu-OMe (**15**), yielding 5.6 g (11.9 mmol, 71% overall) of the desired product **10a** after chromatography on silica gel using ethylacetate and cyclohexane in 1:1 ratio as the eluent. ^1^H NMR (400 MHz, CDCl_3_) δ_H_ ppm: 0.85–0.95 (18H, m), 1.42 (9H, s), 1.51–1.72 (9H, m), 3.70 (3H, s), 4.09 (1H, m), 4.47 (1H, m), 4.54 (1H, m), 4.97 (1H, brs), 6.70 (1H, brs), 6.78 (1H, brs); ^13^C NMR (75 MHz, CDCl_3_) δ_C_ ppm: 21.9, 22.1, 22.1, 22.9, 23.0, 24.7, 24.7, 24.8, 24.8, 28.4, 40.7, 41.1, 41.7, 50.8, 51.5, 52.2, 53.4, 80.0, 155.6, 171.9, 172.9, 173.1; EI-MS *m/z* 456.3 [M − CH_3_], 440.3 [M − OCH_3_]^+^, 398.3 [M − C_4_H_9_O]^+^, 372.3 [M − Boc]^+^, 285.2 [M − C_10_H_20_NO_2_]^+^, 259.2 [M − C_13_H_27_N_2_O_3_]^+^, 186.2 [M − C_14_H_25_N_2_O_4_]^+^.

#### 3.7.4. *N*-Boc-L-Leu-L-Leu-D-Leu-OMe (**10b**)

2.6 g (7.3 mmol) *N*-Boc-L-Leu-L-Leu-OMe (**17**) were saponified following general protocol c. The success of the deprotection step was monitored by ^1^H NMR of the resulting crude product (^1^H data for *N*-Boc-L-Leu-L-Leu: (300 MHz, CDCl_3_) δ_H_ ppm: 0.86–0.99 (12H, m), 1.43 (9H, s), 1.58–1.73 (6H, m), 3.93 (1H, m), 4.62 (1H, m), 5.40 (1H, d, 5.4 Hz), 6.79 (1H, d, 6.8 Hz), 9.03 (1H, brs)). This material was used in the peptide coupling step according to general protocol a with 1.11 g (7.7 mmol) D-Leu-OMe (**18**), yielding 2.9 g (6.15 mmol, 84% overall) of the desired product **10b **after chromatography on silica gel using ethylacetate and cyclohexane in 1:1 ratio as the eluent. ^1^H NMR (400 MHz, CDCl_3_) δ_H_ ppm: 0.88–0.97 (18H, m), 1.44 (9H, s), 1.49–1.78 (9H, m), 3.69 (3H, s), 4.04 (1H, m), 4.44–4.56 (2H, m), 4.89 (1H, d, 6.7 Hz), 6.45 (1H, d, 6.5 Hz), 6.77 (1H, d, 6.8 Hz); ^13^C NMR (75 MHz, CDCl_3_) δ_C_ ppm: 21.7, 21.8, 21.9, 22.1, 22.9, 22.9, 23.0, 24.8, 26.9, 28.3, 40.8, 40.9, 41.0, 50.8. 51.6, 52.1, 53.5, 80.2, 156.0, 172.0, 172.8, 173.2; ESI-MS *m/z* 494.3 [M + Na]^+^, 438.3 [M + Na − C_4_H_9_]^+^, 394.3 [M + Na − C_4_H_9_O]^+^.

#### 3.7.5. *N*-Boc-L-Leu-D-Leu-L-Leu-OMe (**10c**)

3.09 g (8.62 mmol) *N*-Boc-D-Leu-L-Leu-OMe (**14**) were *N*-deprotected following general protocol b1. The success of the deprotection step was monitored by ^1^H NMR of the resulting crude product (^1^H data for D-Leu-L-Leu-OMe: (400 MHz, CDCl_3_) δ_H_ ppm: 0.86–0.95 (12H, m), 1.37 (2H, m), 1.52–1.69 (6H, m), 3.38 (1H, m), 3.69 (3H, s), 4.57 (1H, m) 7.57 (1H, d, 7.6 Hz)). This material was used in the peptide coupling step according to general protocol a with 1.35 g (5.42 mmol) *N*-Boc-L-Leu (**13**), yielding 2.5 g (5.30 mmol, 61% overall) of the desired product **10c** after chromatography on silica gel using ethylacetate and cyclohexane in 1:1 ratio as the eluent. ^1^H NMR (400 MHz, CDCl_3_) δ_H_ ppm: 0.88–0.98 (18H, m), 1.44 (9H, s), 1.49–1.76 (9H, m), 3.70 (3H, s), 4.07 (1H, m), 4.48 (1H, m), 4.54 (1H, m), 4.86 (1H, m), 6.46 (1H, brs), 6.75 (1H, brs); ^13^C NMR NMR (75 MHz, CDCl_3_) δ_C_ ppm: 21.5, 22.4, 22.6, 22.7, 23.0, 24.4, 24.4, 27.9, 29.3, 38.9, 39.9, 40.8, 41.5, 50.4, 51.8, 52.9, 55.6, 79.9, 155.3, 171.4, 172.7, 175.6; ESI-MS *m/z* 494.3 [M + Na]^+^, 438.2 [M + Na − C_4_H_9_]^+^, 394.3 [M + Na − C_4_H_9_O]^+^.

#### 3.7.6. *N*-Boc-L-Leu-L-Leu-l-Leu-OMe (**10d**)

6.51 g (18.17 mmol) *N*-Boc-L-Leu-L-Leu-OMe (**17**) were saponified following general protocol c. The success of the deprotection step was monitored by ^1^H NMR of the resulting crude product (^1^H data for *N*-Boc-L-Leu-L-Leu: see section 3.7.4. This material was used in the peptide coupling step according to general protocol a with 2.67 g (18.3 mmol) L-Leu-OMe (**15**), yielding 6.79 g (14.4 mmol, 79% overall) of the desired product **10d **after chromatography on silica gel using ethylacetate and cyclohexane in 1:1 ratio as the eluent. ^1^H NMR (400 MHz, CDCl_3_) δ_H_ ppm = 0.82–0.92 (18H, m), 1.39 (9H, s), 1.46–1.62 (9H, m), 3.68 (3H,s), 4.11 (1H, m), 4.52 (2H, m), 5.24 (1H, m), 6.82 (1H, m), 7.00 (1H, m); ^13^C NMR (100 MHz, CDCl_3_) δ_C_ ppm: 21.9, 22.0, 22.3, 22.8, 22.9, 24.6, 24.7, 24.8, 24.8, 28.3, 41.01, 41.1, 41.3, 50.7, 51.6, 52.2, 53.0, 79.9, 155.8, 171.9, 172.9, 173.2; ESI-MS *m/z* 965.5 [2M + Na]^+^, 494.3 [M + Na]^+^, 394.3 [M + Na − Boc]^+^.

#### 3.7.7. *N*-Me-L-Leu-OMe (**19**)

6 g (24.06 mmol, 1 equivalent) *N*-Boc-L-Leu (**13**) were dissolved in a mixture of THF and DMF in a 10:1 ratio (198 mL) and treated with MeI (10 mL, 192.54 mmol, 8 equivalent). After cooling down to 0 °C NaH (2.89 g, 72.20 mmol, 3 equivalent, 60% dispersion in oil) was added slowly to the reaction solution. The resulting mixture was stirred at 80 °C under reflux for 20 h. After removing the solvent *in vacuo*, the crude product was taken up in water (150 mL) and extracted with ethyl acetate (3 × 100 mL). The combined organic layers were washed with saturated NaHCO_3_ (2 × 100 mL) and H_2_O (3 × 100 mL) and dried over MgSO_4_. After solvent evaporation the desired product was purified via chromatography on silica gel using ethyl acetate and cyclohexane in 1:1 ratio as the eluent yielding 6.09 g (23.5 mmol, 98%). Treatment of the resulting *N*-Boc-*N*-Me-L-Leu-OMe under conditions described in protocol B1 furnished 3.61 g (22.7 mmol, 94% overall) of product **19**. ^1^H NMR (300 MHz, CDCl_3_) δ_H_ ppm: 0.83 (6H, m), 1.36–1.39 (3H, m), 1.57–1.64 (1H, m), 2.27 (3H, s), 3.11 (1H, m), 3.64 (3H, s); ^13^C NMR (75 MHz, CDCl_3_) δ_C_ ppm: 22.3, 22.6, 24.9, 34.6, 42.5, 51.5, 61.8, 176.2; ESI-MS *m/z* 182.1[M + Na]^+^, 160.1 [M + H]^+^, 100.1 [M − CO_2_Me]^+^.

#### 3.7.8. *N*-Boc-L-Val-N-Me-L-Leu (**11**)

1.40 g (8.80 mmol) *N*-Me-L-Leu-OMe (**19**)were dissolved in CH_2_Cl_2_ (40 mL) and HOBt, EDC and DIPEA were added successively to the solution which was stirred at 0 °C for 20 min. 1.95 g (9.96 mmol) *N*-Boc-L-Val (**12**) in CH_2_Cl_2_ (40 mL) was added to the reaction mixture and it was stirred at room temperature overnight. After quenching with saturated NH_4_Cl solution, the aqueous layer was extracted with CH_2_Cl_2_. The combined organic layers were washed with saturated NH_4_Cl solution and dried over MgSO_4_. After solvent evaporation the product was purified via chromatography on silica gel using ethyl acetate and cyclohexane in 1:1 ratio, yielding 2.14 g (5.97 mmol, 68%) of the desired coupling product *N*-Boc-L-Val-*N*-Me-L-Leu-OMe. ^1^H NMR (300 MHz, CDCl_3_) δ_H_ ppm: 0.88–0.95 (12H, m), 1.41 (9H, s), 1.62–1.71 (3H, m), 1.99 (1H, m), 3.00 (3H, s), 3.68 (3H, s), 4.41 (1H, m), 5.21 (1H, m), 5.36 (1H, m); ^13^C NMR (75 MHz, CDCl_3_) δ_C_ ppm: 17.7, 19.4, 21.5, 23.4, 24.8, 28,4, 29.8, 31.2, 37.1, 52.2, 54.5, 55.5, 79.6, 156.1, 172.3, 173.6; ESI-MS *m/z* (%) = 381.3 [M + Na]^+^, 367.2 [M + Na − CH_3_]^+^, 281.2 [M − C_4_H_9_O_2_]^+^. Saponification of the latter following protocol c delivered 1.8 g (5.13 mmol, 58% overall) of the dipeptide building block 11. ^1^H NMR (300 MHz, CDCl_3_) δ_H_ ppm: 0.88–0.98 (12H, m), 1.41 (9H, s), 1.74 (3H, m), 1.99 (1H, m), 3.05 (3H, s), 4.41 (1H, m), 5.36 (1H, d, 5.2 Hz), 5.48 (1H, m), 8.54 (1H, brs); ^13^C NMR (75 MHz, CDCl_3_) δ_C_ ppm: 17.9, 19.4, 21.5, 23.4, 24.8, 28.4, 29.8, 31.7, 37.0, 54.8, 55.7, 79.7, 156.3, 174.3, 176.1; ESI-MS *m/z* 367.2 [M + Na]^+^, 353.2 [M + Na − CH_3_]^+^.

#### 3.7.9. *N*-Boc-L-Val-*N*-Me-L-Leu-D-Leu-L-Leu-L-Leu-OMe (**20a**)

3.00 g (6.36 mmol) *N*-Boc-D-Leu-L-Leu-L-Leu-OMe (**10a**) were *N*-deprotected following general protocol b1. The success of the deprotection step was monitored by ^1^H NMR of the resulting crude product (^1^H data for D-Leu-L-Leu-L-Leu-OMe: (400 MHz, DMSO-*d*_6_) δ_H_ ppm: 0.82–0.89 (18H, m), 1.23 (1H, m), 1.44–1.63 (8H, m), 3.22 (2H, m), 3.59 (3H, s), 3.22 (1H, m), 4.26 (1H, m), 4.45 (1H, m), 8.01 (1H, m), 8.26 (1H, d, 8.3 Hz)). This material was used in the peptide coupling step according to general protocol a with 1.02 g (4.55 mmol) *N*-Boc-L-Val-*N*-Me-L-Leu (**11**), yielding 2.17 g (3.11 mmol, 49% overall) of the desired product **20a** after chromatography on silica gel using ethyl acetate and cyclohexane in 1:1 ratio as the eluent. ESI-MS *m/z* 720.4 [M + Na]^+^, 706.4 [M + Na − CH_3_]^+^.

#### 3.7.10. *N*-Boc-L-Val-*N*-Me-L-Leu-L-Leu-L-Leu-D-Leu-OMe (**20b**)

3.51 g (7.44 mmol) *N*-Boc-L-Leu-L-Leu-D-Leu-OMe (**10b**) were *N*-deprotected following general protocol b1. The success of the deprotection step was monitored by ^1^H NMR of the resulting crude product (^1^H data for L-Leu-L-Leu-D-Leu-OMe: (400 MHz, CDCl_3_-*d*_6_) δ_H_ ppm: 0.88–0.98 (18H, m), 1.34 (1H, m), 1.50–1.77 (8H, m), 3.46 (1H, m), 3.69 (3H, s), 4.44 (1H, m), 4.54 (1H, m), 6.80 (1H, d, 8.0 Hz), 7.66 (1H, d, 8.4 Hz)). This material was used in the peptide coupling step according to general protocol a with 1.26 g (6.57 mmol) *N*-Boc-L-Val-*N*-Me-L-Leu (**11**), yielding 2.94 g (4.21 mmol, 57% overall) of the desired product **20b** after chromatography on silica gel using ethyl acetate and cyclohexane in 1:1 ratio as the eluent. ESI-MS *m/z* 720.5 [M + Na]^+^, 706.5 [M + Na − CH_3_]^+^, 370.2 [C_19_H_36_N_3_O_4_], 327.2 [C_17_H_32_N_2_O_4_]^+^.

#### 3.7.11. *N*-Boc-L-Val-*N*-Me-L-Leu-L-Leu-d-Leu-L-Leu-OMe (**20c**)

1.91 g (4.04 mmol) *N*-Boc-L-Leu-D-Leu-L-Leu-OMe (**10c**) were *N*-deprotected following general protocol b2. The success of the deprotection step was monitored by ^1^H NMR of the resulting crude product (^1^H data for L-Leu-D-Leu-L-Leu-OMe: (400 MHz, DMSO-*d*_6_) δ_H_ ppm: 0.81–0.90 (18H, m), 1.45–1.71 (9H, m), 3.60 (3H, s), 3.82 (1H, m), 4.30 (1H, m), 4.37 (1H, m), 8.35 (2H, m), 8.52 (1H, d, 8.5 Hz), 8.89 (1H, d, 8.9 Hz)). This material was used in the peptide coupling step according to general protocol a with 0.41 g (1.81 mmol) *N*-Boc-L-Val-*N*-Me-L-Leu (**11**), yielding 0.94 g (1.35 mmol, 33% overall) of the desired product **20c** after chromatography on silica gel using ethyl acetate and cyclohexane in 1:1 ratio as the eluent. ESI-MS *m/z* 1418.0 [2M + Na]^+^, 720.5 [M + Na]^+^, 706.5 [M + Na − CH_3_]^+^, 698.5 [M + H]^+^, 598.5 [M − Boc − H]^+^, 553.4 [M − C_7_H_14_NO_2_]^+^, 440.3 [M − C_13_H_25_N_2_O_3_]^+^, 327.2 [M − C_19_H_36_N_3_O_4_], 370.2 [C_19_H_36_N_3_O_4_].

#### 3.7.12. *N*-Boc-L-Val-*N*-Me-L-Leu-L-Leu-L-Leu-L-Leu-OMe (**20d**)

3.99 g (8.46 mmol) *N*-Boc-L-Leu-L-Leu-L-Leu-OMe (**10d**) were *N*-deprotected following general protocol b2. The success of the deprotection step was monitored by ^1^H NMR of the resulting crude product (^1^H data for L-Leu-L-Leu-L-Leu-OMe: (400 MHz, DMSO-*d*_6_) δ_H_ ppm: 0.81–0.93 (18H, m), 1.46–1.66 (9H, m), 3.60 (3H, s), 3.77 (1H, m), 4.30 (1H, m), 4.41 (1H, m), 8.28 (2H, brs), 8.45 (1H, d, 7.8 Hz), 8.67 (1H, d, 8.3 Hz)). This material was used in the peptide coupling step according to general protocol a with 2.75 g (12.26 mmol) *N*-Boc-L-Val-*N*-Me-L-Leu (**11**), yielding 4.5 g (6.45 mmol, 76% overall) of the desired product **20d** after chromatography on silica gel using ethyl acetate and cyclohexane in 1:1 ratio as the eluent. ESI-MS *m/z* 720.6 [M + Na]^+^, 706.5 [M + Na − CH_3_]^+^.

#### 3.7.13. Lajollamide B (**7**)

1.79 mg (2.56 mmol) *N*-Boc-l-Val-*N*-Me-L-Leu-D-Leu-L-Leu-L-Leu-OMe (**20a**) were saponified following general protocol c. The resulting product was dissolved in CH_2_Cl_2_ and PFP, EDC and DMAP were added following general protocol d. After the *N*-deprotection following general protocol b2 the resulting crude product was further used without further purification. A solution of 1.62 g (2.06 mmol) of the linear precursor in CH_2_Cl_2_ (350 mL) and a solution of NEt_3_ (4.7 mL, 33.6 mmol) in CH_2_Cl_2_ (240 mL) were conducted following general protocol d, yielding 0.94 g (1.66 mmol, 65% overall) of lajollamide B. ^1^H NMR (300 MHz, CDCl_3_) δ_H_ ppm: 0.90–0.98 (30H, m), 1.42–1.63 (6H, m), 1.70 (6H, m), 2.28 (1H, m), 2.86 (3H, s), 3.33 (1H, brs), 4.31 (1H, m), 4.47 (1H, m), 4.63 (1H, m), 4.70 (1H, m), 5.00 (1H, m), 7.08 (1H, brs), 8.00 (1H, brs), 8.30 (1H, brs); ^13^C NMR (300 MHz, CDCl_3_) δ_C_ ppm: 18.8, 19.7, 21.8, 22.4, 22.6, 22.7, 22.9, 23.1, 23.3, 23.4, 24.9, 25.1, 25.2, 25.5, 29.8, 36.7, 37.7, 39.7, 39.9, 43.2, 50.8, 51.4, 54.0, 54.1, 58.2, 168.5, 171.5, 171.9, 172.6, 173.9; ESI-MS *m/z* 588.4 [M + Na]^+^, 574.4 [M + Na − CH_3_]^+^, 560.4 [M + Na − C_4_H_16_]^+^.

#### 3.7.14. Lajollamide C (**8**)

270 mg (0.39 mmol) of *N*-Boc-L-Val-*N*-Me-L-Leu-L-Leu-L-Leu-d-Leu-OMe (**20b**) were saponified following general protocol c. The resulting product was dissolved in CH_2_Cl_2_ and PFP, EDC and DMAP were conducted following general protocol d. After *N*-deprotection following protocol b2 the resulting crude product was used without further purification. A solution of 180 mg (0.23 mmol) of the linear precursor in CH_2_Cl_2_ (80 mL) and a solution of NEt_3_ (0.6 mL, 4.58 mmol) in CH_2_Cl_2_ (25 mL) were conducted following general protocol d, yielding 120 mg (0.012 mmol, 54% overall) of lajollamide C. ^1^H NMR (400 MHz, CDCl_3_) δ_H_ ppm: 0.88–0.97 (30H, m), 1.46–1.76 (12H, m), 2.00 (1H, m), 3.13 (3H, brs), 4.36 (1H, m), 4.47 (1H, m), 4.58–4.63 (2H, m), 4.79 (1H, m), 6.72 (1H, brs), 7.01 (2H, brs), 7.43 (1H, brs); ^13^C NMR (400 MHz, CDCl_3_) δ_C_ ppm: 18.7, 19.2, 21.8, 22.4, 22.4, 22.5, 22.8, 22.8, 23.0, 23.1, 25.0, 25.1, 25.2, 25.3, 31.0, 31.5, 37.1, 38.2, 40.3, 41.4, 51.0, 52.2, 53.0, 55.3, 55.6, 170.5, 171.7, 172.0, 172.2, 174.4; ESI-MS *m/z* 588.4 [M + Na]^+^, 566.4 [M + H]^+^.

#### 3.7.15. Lajollamide D (**9**)

0.19 g (0.27 mmol) of *N*-Boc-L-Val-*N*-Me-L-Leu-L-Leu-d-Leu-L-Leu-OMe (**20c**) were saponified following general protocol c, yielding 0.14 g (0.20 mmol, 75%) of the desired product. This was dissolved in CH_2_Cl_2_ and PFP, EDC and DMAP were conducted following general protocol d. After *N*-deprotection the following general protocol b2 resulting crude product was used without further purification. 80 mg (0.11 mmol) of the linear precursor in CH_2_Cl_2_ (37 mL) and a solution of NEt_3_ (0.3 mL, 2.13 mmol) in CH_2_Cl_2_ (12.5 mL) were conducted following general protocol d, yielding 40 mg (0.07 mmol, 26% overall) of lajollamide D. ^1^H NMR (300 MHz, CDCl_3_) δ_H_ ppm: 0.86–0.94 (30H, m), 1.50–1.86 (12H, m), 2.13 (1H, m), 3.03 (3H, s), 3.51 (1H, m), 4.20 (2H, m), 4.47 (2H, m), 6.42 (1H, brs), 6.58 (1H, brs), 7.10 (1H, brs), 8.19 (1H, d, 8.2 Hz); ^13^C NMR (300 MHz, CDCl_3_) δ_C_ ppm: 18.1, 19.9, 21.3, 22.0, 22.4, 22.4, 22.7, 22.8, 23.0, 23.3, 24.9, 25.0, 25.4, 25.8, 30.5, 38.4, 39.6, 40.0, 40.1, 41.0, 51.1, 52.8, 54.2, 55.9, 70.3, 171.7, 171.9, 172.0, 172.7, 173.4; ESI-MS *m/z* 588.4 [M + Na]^+^, 574.4 [M + Na − CH_3_]^+^, 560.4 [M + Na − C_4_H_16_]^+^.

#### 3.7.16. Lajollamide A (**1**)

4.5 g (6.45 mmol) of *N*-Boc-L-Val-*N*-Me-L-Leu-L-Leu-L-Leu-L-Leu-OMe (**20d**) was saponified following the general protocol c. The resulting product was dissolved in CH_2_Cl_2_ and PFP, EDC and DMAP were added following the general procedure d. After *N*-deprotection following protocol b2 the resulting crude product was used without further purification. A solution of 4g (5.09 mmol) of the linear precursor in CH_2_Cl_2_ (500 mL) and a solution of NEt_3_ (14 mL, 101.74 mmol) in CH_2_Cl_2_ (200 mL) were conducted following general protocol d, yielding 1.73 g (3.06 mmol, 47% overall) of lajollamide A. ^1^H NMR (400 MHz, CDCl_3_) δ_H_ ppm: 0.88–1.00 (30H, m), 1.51–1.64 (9H, m), 1.89 (3H, m), 2.22 (1H, m), 3.36 (3H, s), 3.51 (1H, brs), 4.25 (1H, m), 4.47 (1H, m), 4.58 (2H, m), 6.04 (1H, brs), 6.65 (1H, brs), 6.84 (1H, brs), 7.38 (1H, brs); ^13^C NMR (300 MHz, CDCl_3_) δ_C_ ppm: 18.6, 19.3, 21.4, 21.9, 22.1, 22.4, 22.8, 23.0, 23.4, 23.6, 24.8, 25.1, 25.4, 25.5, 30.4, 37.2, 37.6, 40.1, 40.8, 41.1, 50.4, 51.4, 53.9, 55.5, 65.3, 171.6, 172.2, 172.7, 173.3, 173.9; ESI-MS *m/z* 588.4 [M + Na]^+^, 566.4 [M + H]^+^.

### 3.8. Biological Activity

The antimicrobial activities of compounds **1**, **7**, **8** and **9** were determined as previously described [34] using the following indicator strains: *Candida albicans* (DSM 1386), *Bacillus subtilis* (DSM 347), *E. coli* K12 (DSM 498), *Xanthomonas campestris* (DSM 2405), *Staphylococcus epidermidis* (DSM 20044) and methicillin-resistant *Staphylococcusaureus* (MRSA) (DSM 18827). Furthermore, the compounds were tested for inhibition of phosphodiesterase (PDE4-4B2) and cytotoxicity to HepG2(human hepatocellular carcinoma) cells. The determination of the acetylcholinesterase inhibitory activity was performed according to Ohlendorf *et al.* (2012) [35]. Glycogen synthase kinase-3β inhibition was determined as described by Baki *et al.* (2007) [36]. The activity of compounds **4** and **6** was tested in agar diffusion assays against the bacteria *Escherichiacoli* and *Bacillus megaterium*, the fungi *Microbotryum violoaceum*, *Eurotium repens* and *Mycothypha microsporum*, and the green microalga *Chlorella fusca*, as previously described [37]. Benzylpenicillin (6 mm growth inhibition towards *Bacillus megaterium*) and streptomycin (5 mm and 10 mm total inhibition towards *Escherichia coli* and *Bacillus megaterium*, respectively) were used as positive control for the antibacterial agar diffusion assays. In antifungal and antialgal assays, miconazole served as a positive control, resulting in total inhibition zones of 6–10 mm.

## 4. Conclusions

In this paper we described the isolation and full structural characterization of the antibacterial marine fungus-derived cyclopentapeptide lajollamide A (**1**). Stereochemical investigations were successfully completed by total synthesis of **1**, along with three unnatural congeners, lajollamides B–D (**7**–**9**). The unexpected epimerization of Leu units in the lajollamide framework observed during our studies clearly demonstrates the danger in misassigning natural product structures elucidated by spectroscopic methods and relatively harsh analytical techniques alone. This demonstrates the importance of total synthesis for the unambiguous structural assignment of new molecules. 

It is interesting to note that cyclopentapeptides structurally related to lajollamide A (**1**) seem to be widespread among fungal strains. Examples include the cytotoxic sansalvamide [*cyclo*-(*O*-Leu-Val-Leu-Phe-Leu)] from a marine *Fusarium* strain [38], a cyclic pentapeptide recently isolated by Laatsch and coworkers [*cyclo*-(Ile-Leu-Leu-Leu-Leu)] from an endophytic fungus *Cryptosporiopsis* sp. [39], and a compound described by Li *et al.* [*cyclo*-(Phe-Leu-Leu-Leu-Leu)] [40] These metabolites share a Leu-rich backbone incorporating one or two differing amino acids with likewise hydrophobic (Val, Ile) or aromatic (Phe) side-chains. In addition, similar compounds can also be found in bacteria, e.g., the game-X-peptides [e.g., *cyclo*-(L-Leu-D-Phe-D-Leu-Leu-D-Val)] from *Photorhabdus luminescens*, albeit with D-configured amino acids being incorporated into these compounds via their NRPS-mediated biosynthesis [41]. The broad occurrence of such peptides clearly raises as yet unanswered questions about their true function and importance in ecological systems.

Besides the characterization of lajollamide **A** (**1**), this work provided further insights into the secondary metabolome of an underexplored marine fungal isolate *Asteromyces cruciatus*. This organism proved capable of producing a series of other biomedically interesting natural products, compounds **2–6**. The biological properties of all isolated compounds were investigated in a series of assay systems, revealing the lack of any cytotoxic or enzyme inhibitory activities in the biological tests used, but antibiotic properties of 1 and **6–9**. The work presented here thus expands the small number of biomedically interesting metabolites known from *A. cruciatus* and further demonstrates the high potential of marine fungi for the production of diverse biologically active small molecules.

## Supplementary Files

Supplementary File 1
