# Supplementary material for: Isolation, Structure Elucidation and Total Synthesis of Lajollamide A from the Marine Fungus Asteromyces cruciatus
Source: Mar Drugs. 2012 Dec 19;10(12):2912–35. doi: 10.3390/md10122912 (PMC3528133; doi:10.3390/md10122912)

Supplementary Information

Figure S1. ESI-MS and HR-ESI-MS spectrum of Lajollamide A (**1**). 2

Figure S2. 300 MHz ^{1}H NMR spectrum of Lajollamide A (**1**). 3

Figure S3. 75 MHz ^{13}C NMR and DEPT135 NMR spectra of Lajollamide A (**1**). 4

Figure S4. 300 MHz HSQC NMR spectrum of Lajollamide A (**1**). 5

Figure S5. 300 MHz ^{1}H–^{1}H–COSY NMR spectrum of Lajollamide A (**1**). 6

Figure S6. 300 MHz ^{1}H–^{13}C–HMBC NMR spectrum of Lajollamide A (**1**). 7

Figure S7. 300 MHz ^{1}H NMR and 75 MHz ^{13}C NMR spectra of Regiolone (**2**). 8

Figure S8. 300 MHz ^{1}H NMR and 75 MHz ^{13}C NMR spectra of (3*R*,6*R*)-Hyalodendrin (**3**). 9

Figure S9. 300 MHz ^{1}H NMR and 75 MHz ^{13}C NMR spectra of (3*R*,6*R*)-Gliovictin (**4**). 10

Figure S10. 300 MHz ^{1}H NMR and 75 MHz ^{13}C NMR spectra of (3*R*,6*R*)-^{1}*N*-Norgliovictin (**5**). 11

Figure S11. 300 MHz ^{1}H NMR and 75 MHz ^{13}C NMR spectra of (3*R*,6*R*)-bis-*N*-Norgliovictin (**6**). 12

Figure S1. ESI-MS and HR-ESI-MS spectrum of **1**.

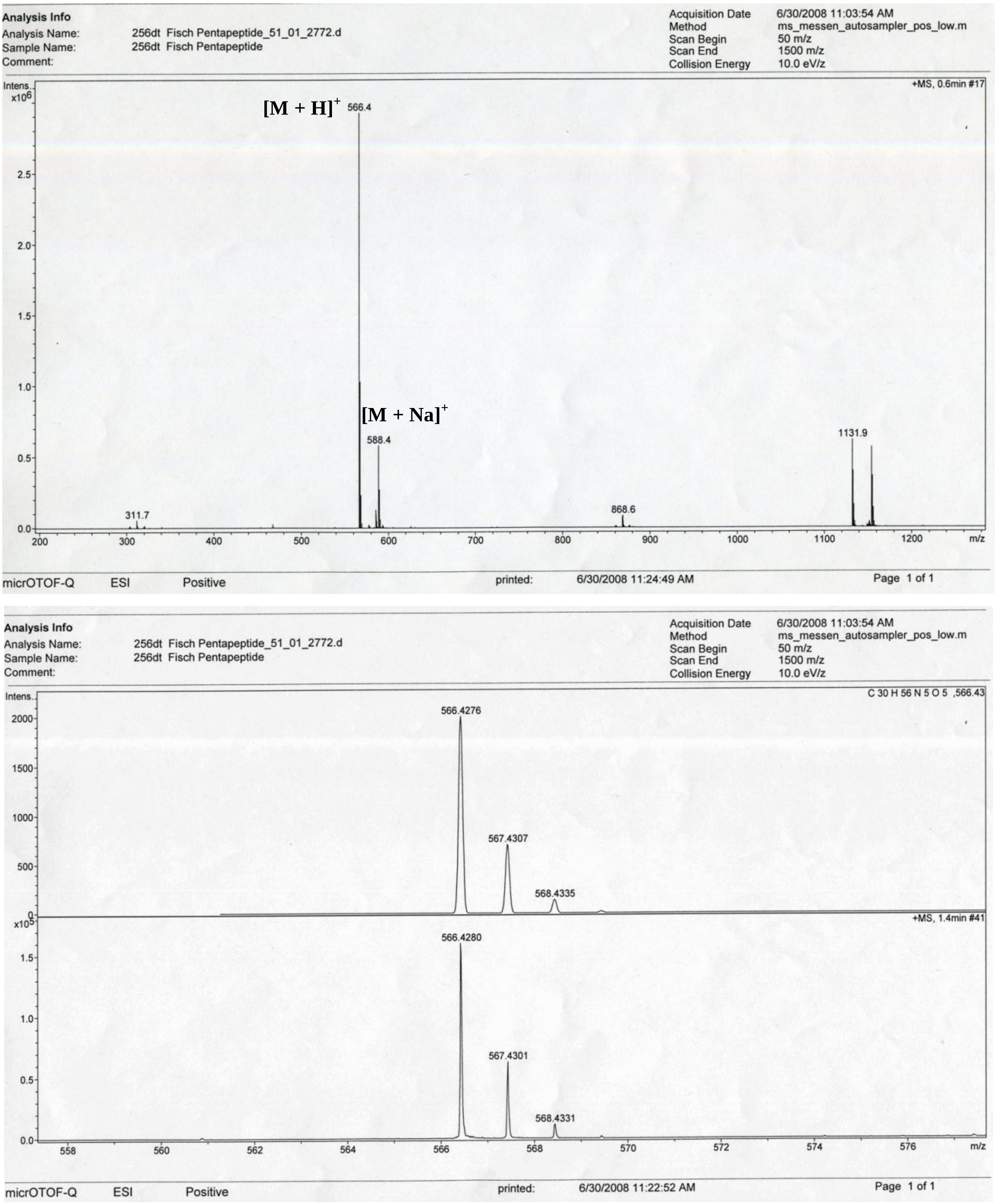

Figure S2. 300 MHz ^{1}H NMR spectrum of **1** in $CDCl_3$.

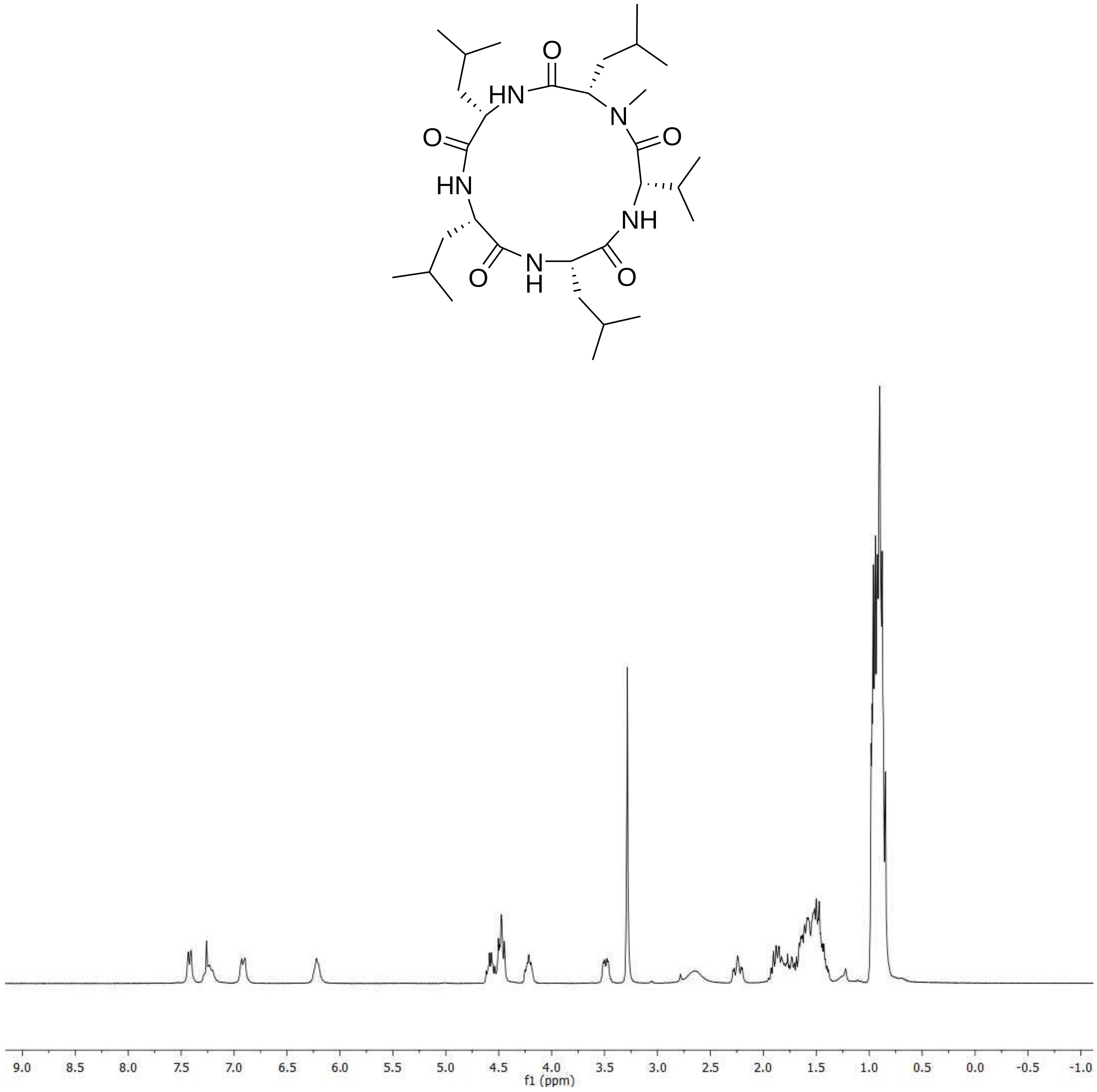

Figure S3. (**A**) 75 MHz ^{13}C NMR spectrum of **1** in $CDCl_3$; (**B**) DEPT135 NMR spectrum of **1** in $CDCl_3$.

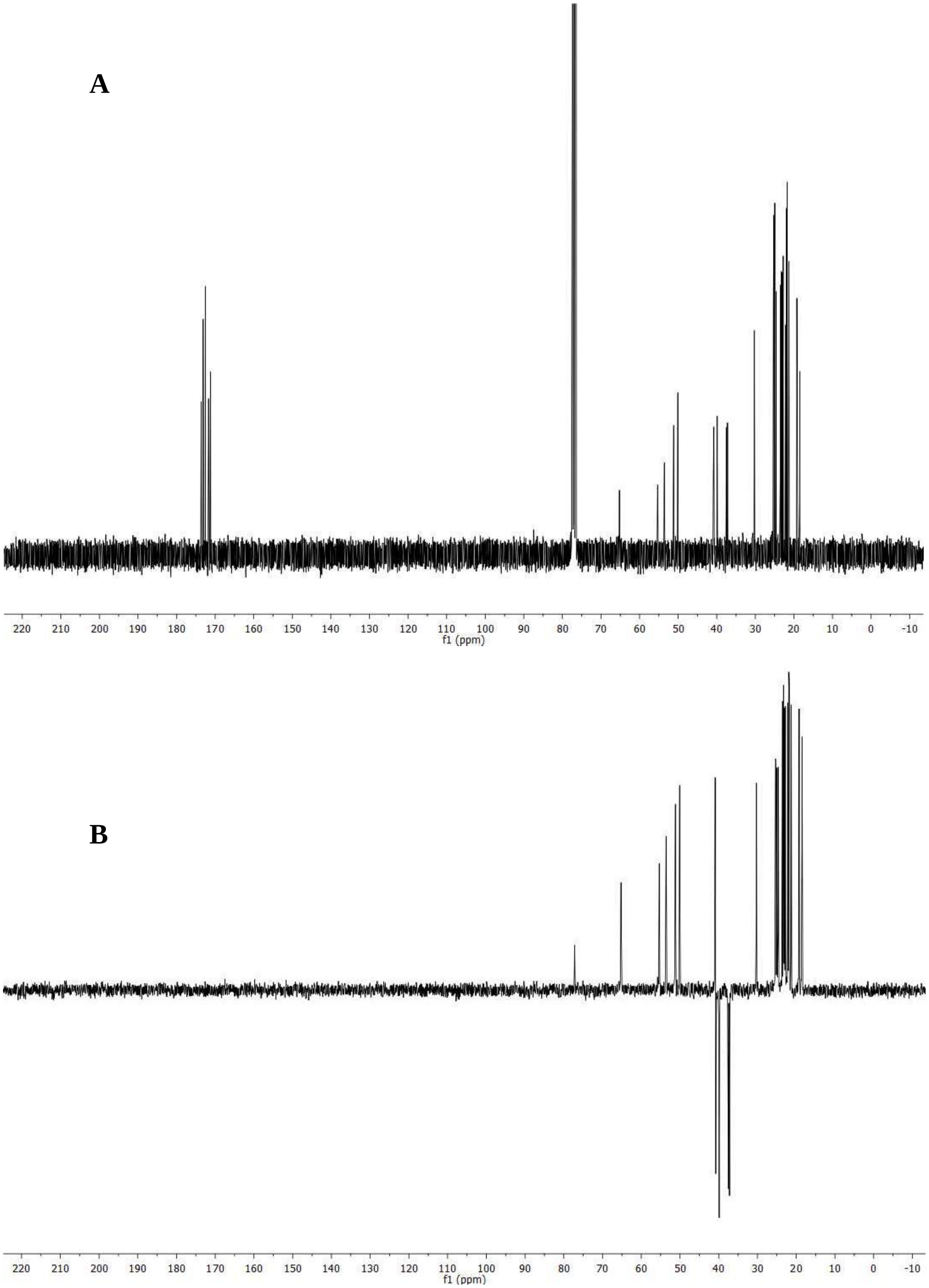

Figure S4. 300 MHz HSQC NMR spectrum of **1** in $CDCl_3$.

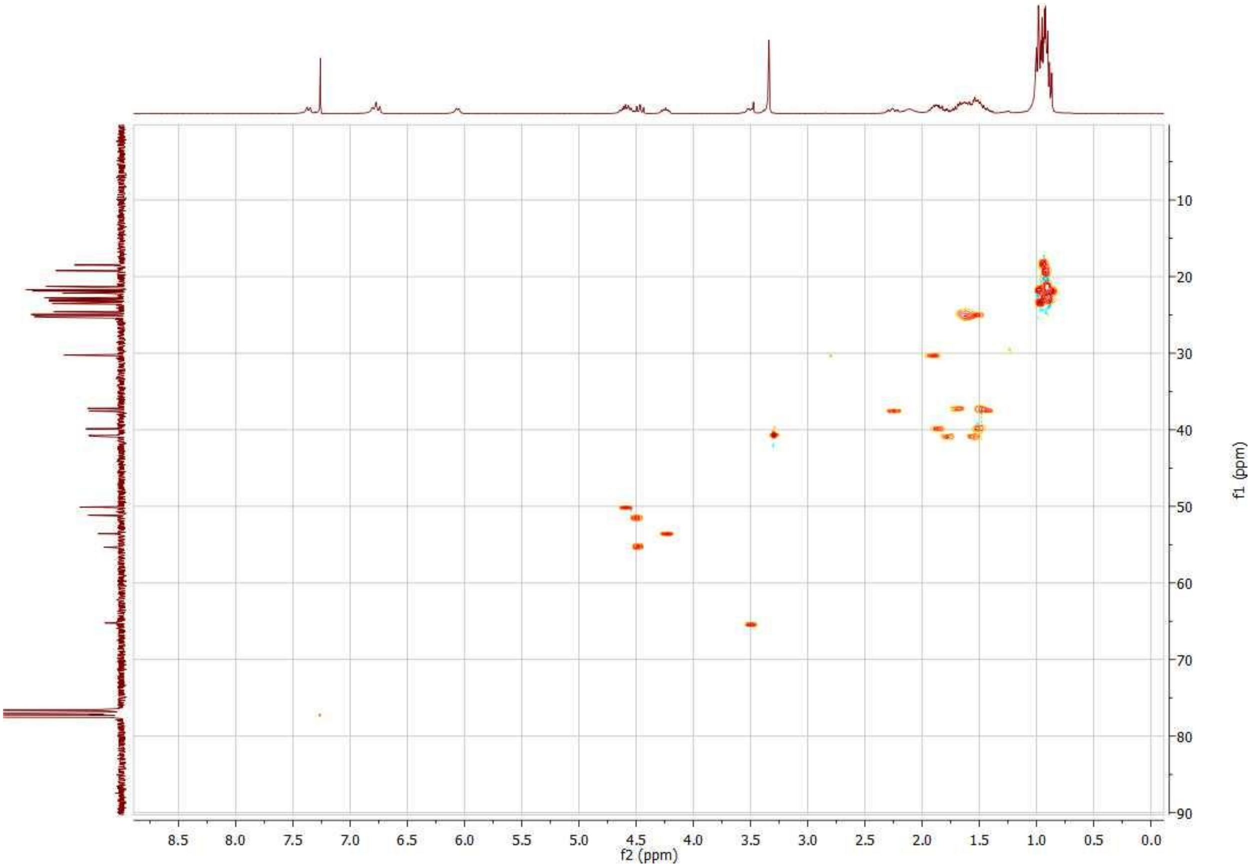

Figure S5. 300 MHz ^{1}H–^{1}H COSY NMR spectrum of **1** in $CDCl_3$.

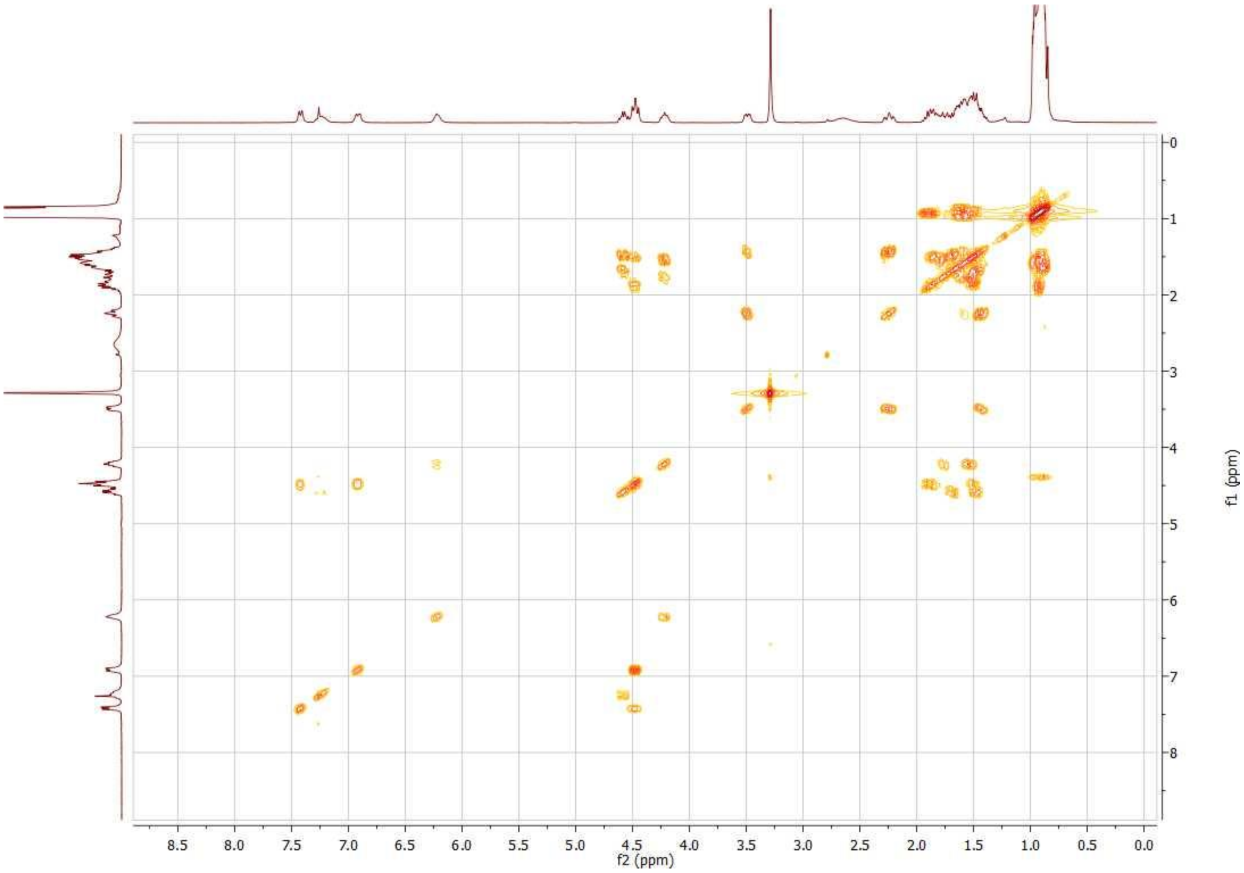

Figure S6. 300 MHz ^{1}H–^{13}C HMBC NMR spectrum of **1** in $CDCl_3$.

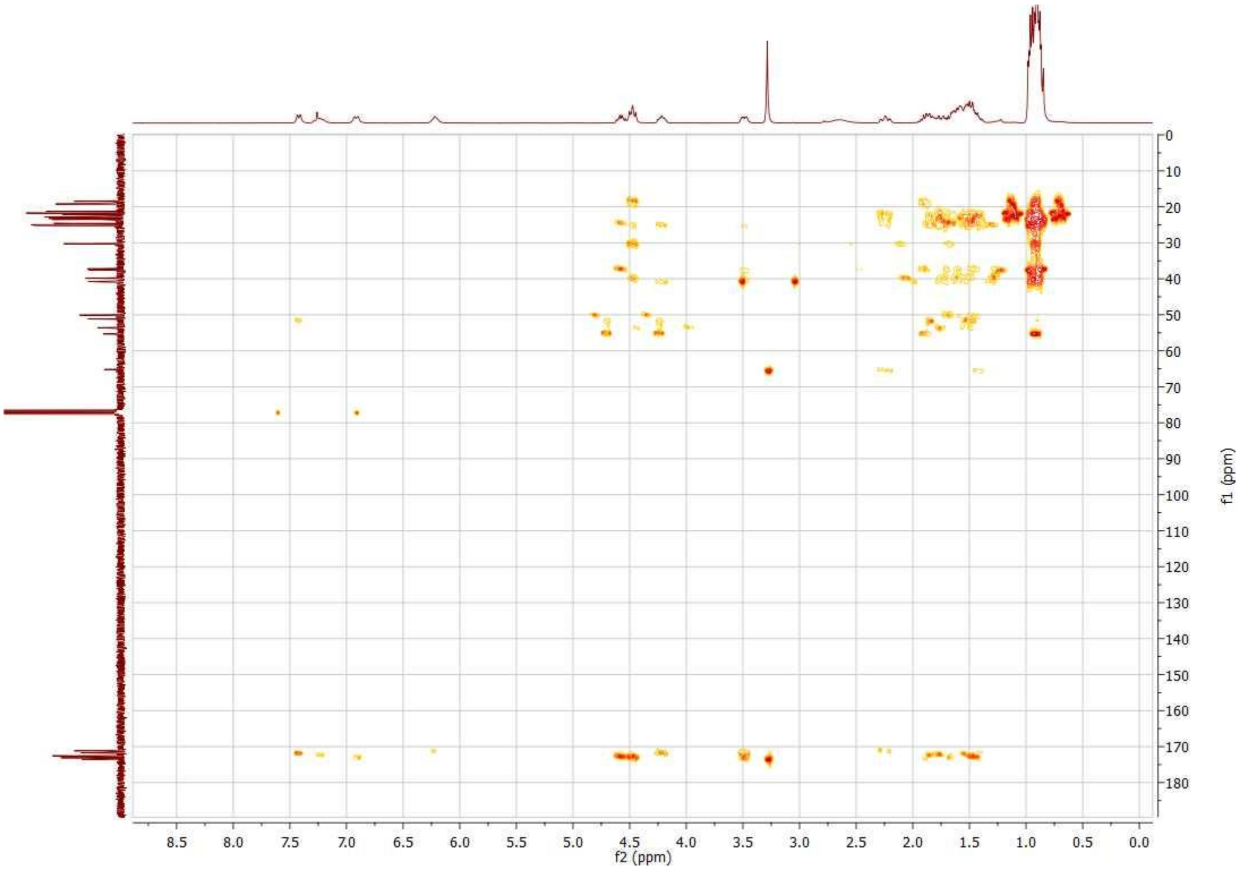

Figure S7. (**A**) 300 MHz ^{1}H NMR spectrum of **2** in $CDCl_3$; (**B**) 75 MHz ^{13}C NMR spectrum of **2** in $CDCl_3$.

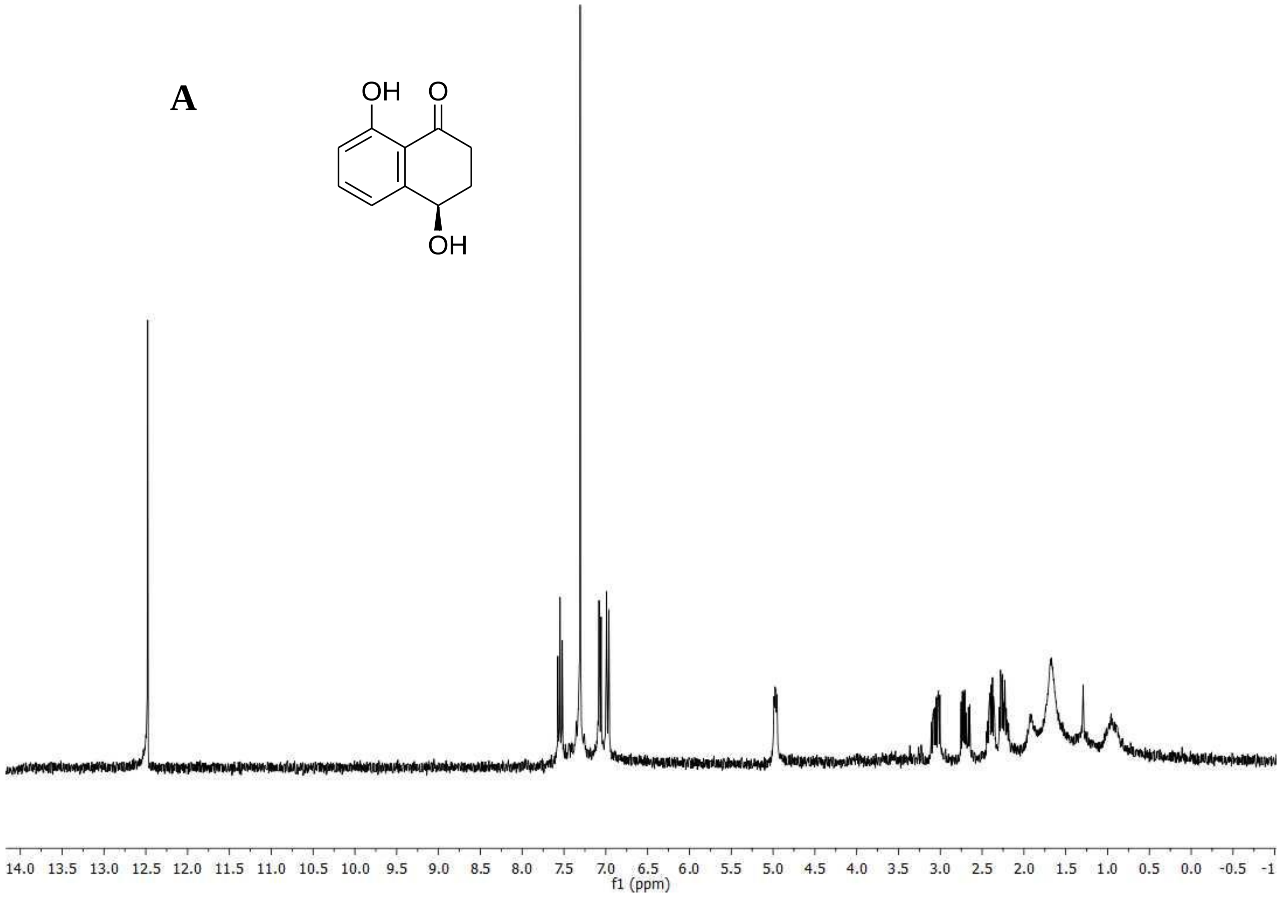

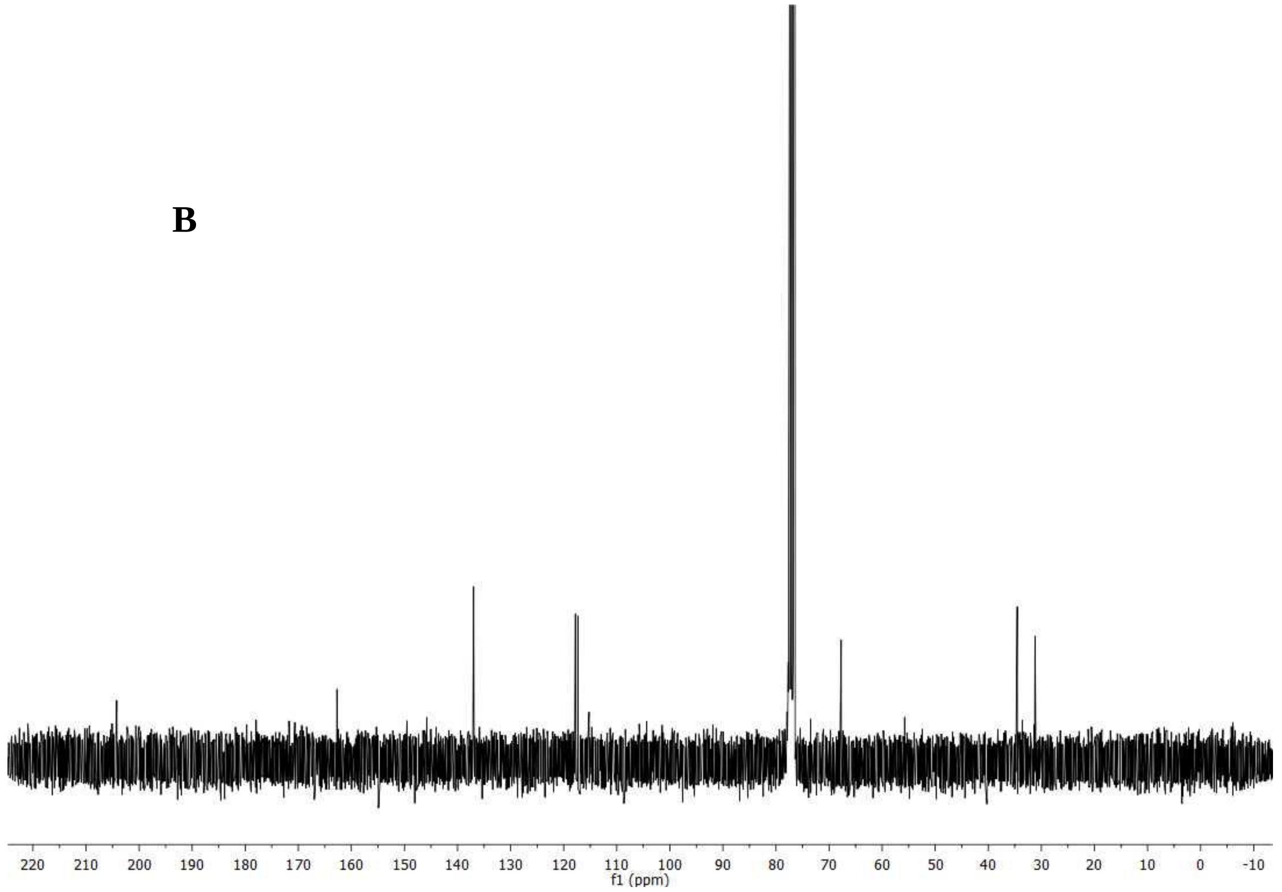

Figure S8. (**A**) 300 MHz ^{1}H NMR spectrum of **3** in $CDCl_3$; (**B**) 75 MHz ^{13}C NMR spectrum of **3** in $CDCl_3$.

A

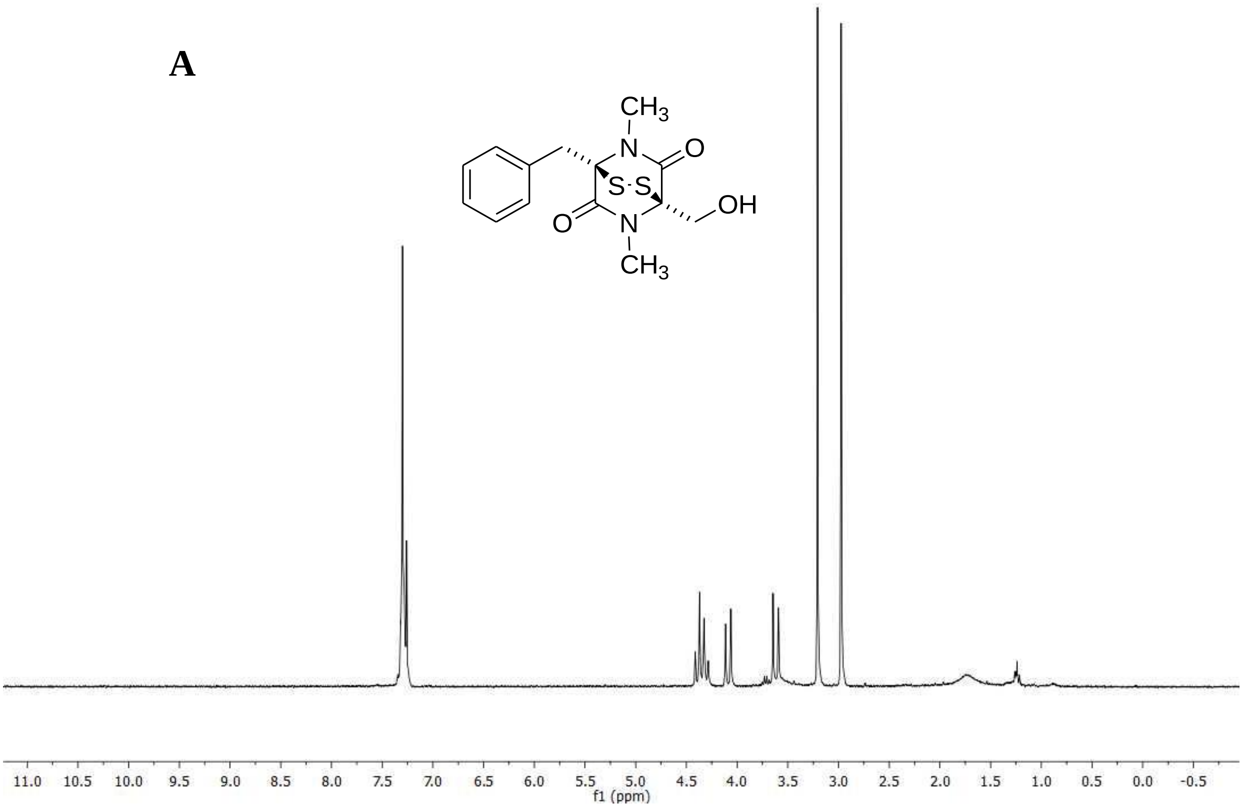

B

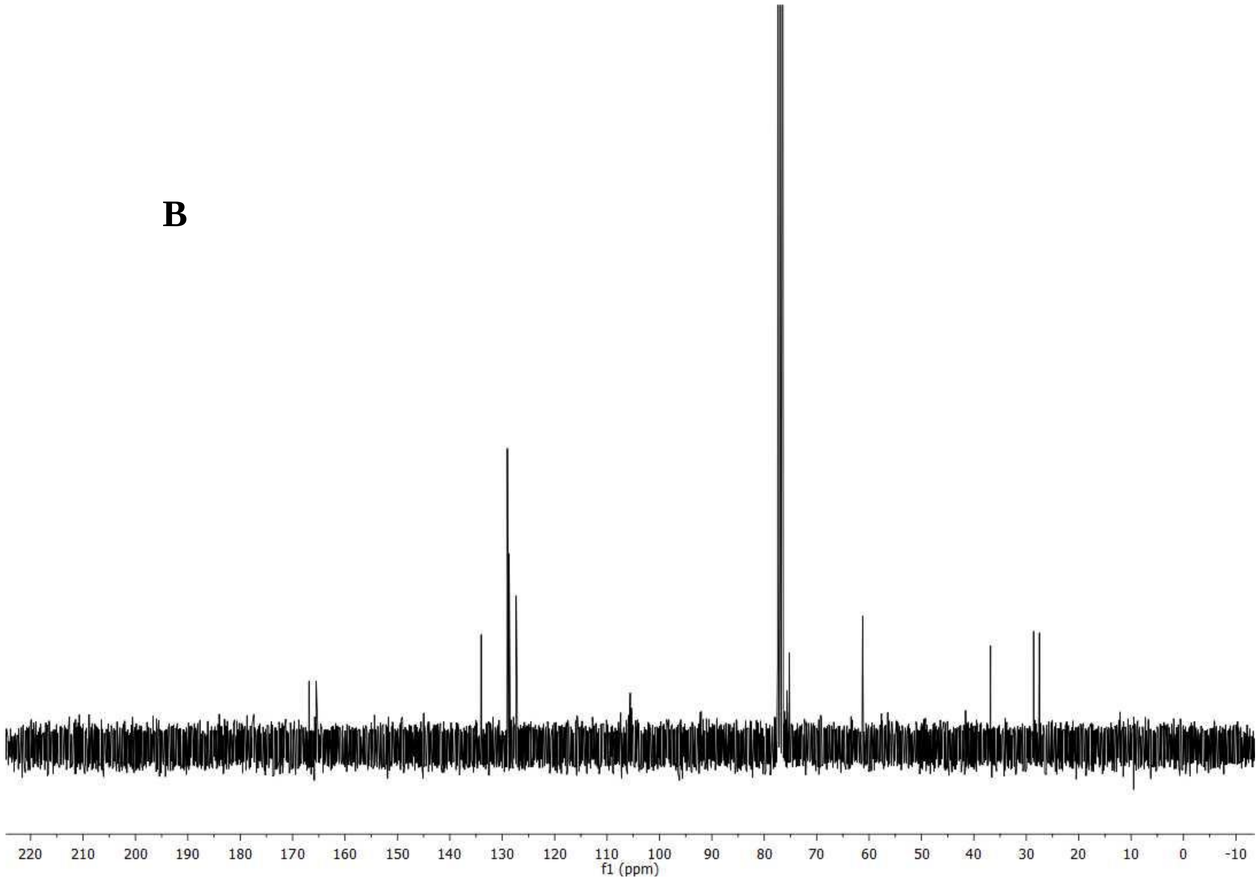

Figure S9. (**A**) 300 MHz ^{1}H NMR spectrum of **4** in $CDCl_3$; (**B**) 75 MHz ^{13}C NMR spectrum of **4** in $CDCl_3$.

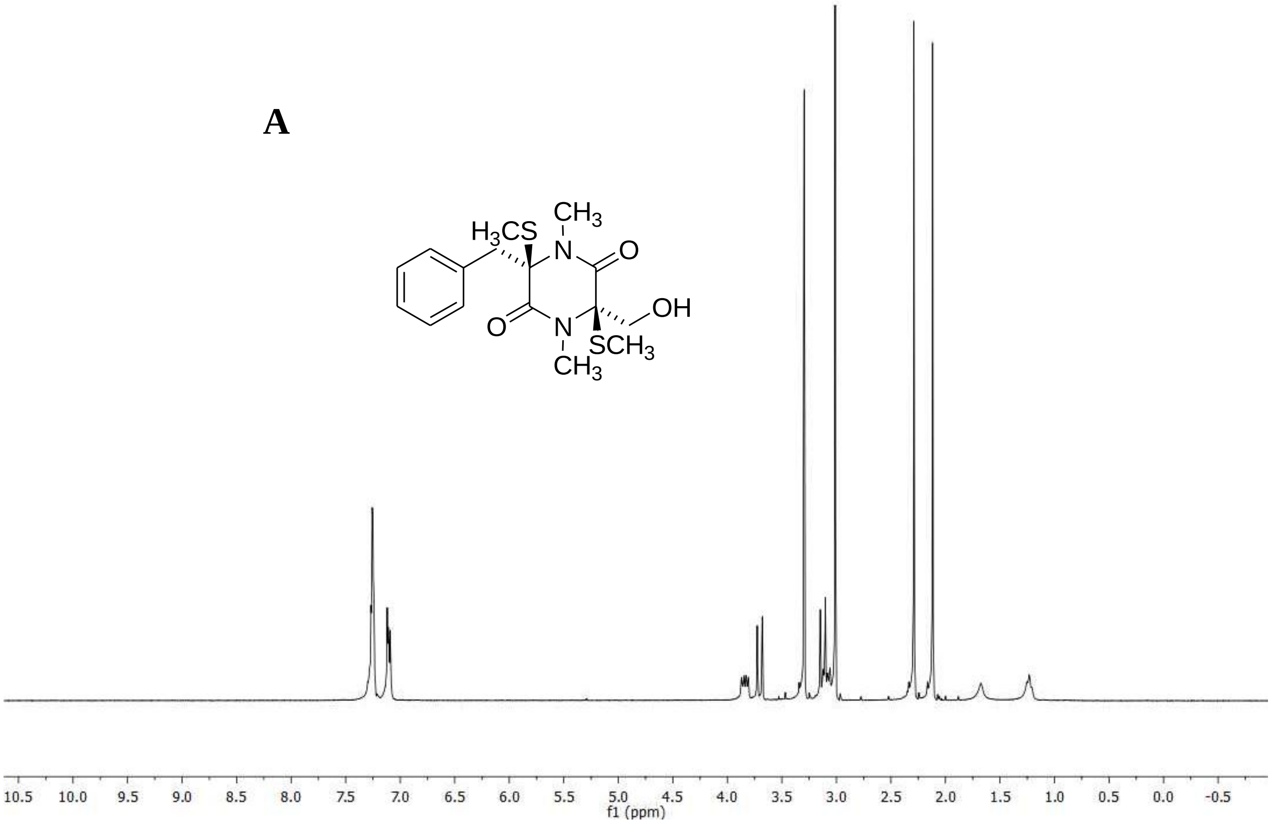

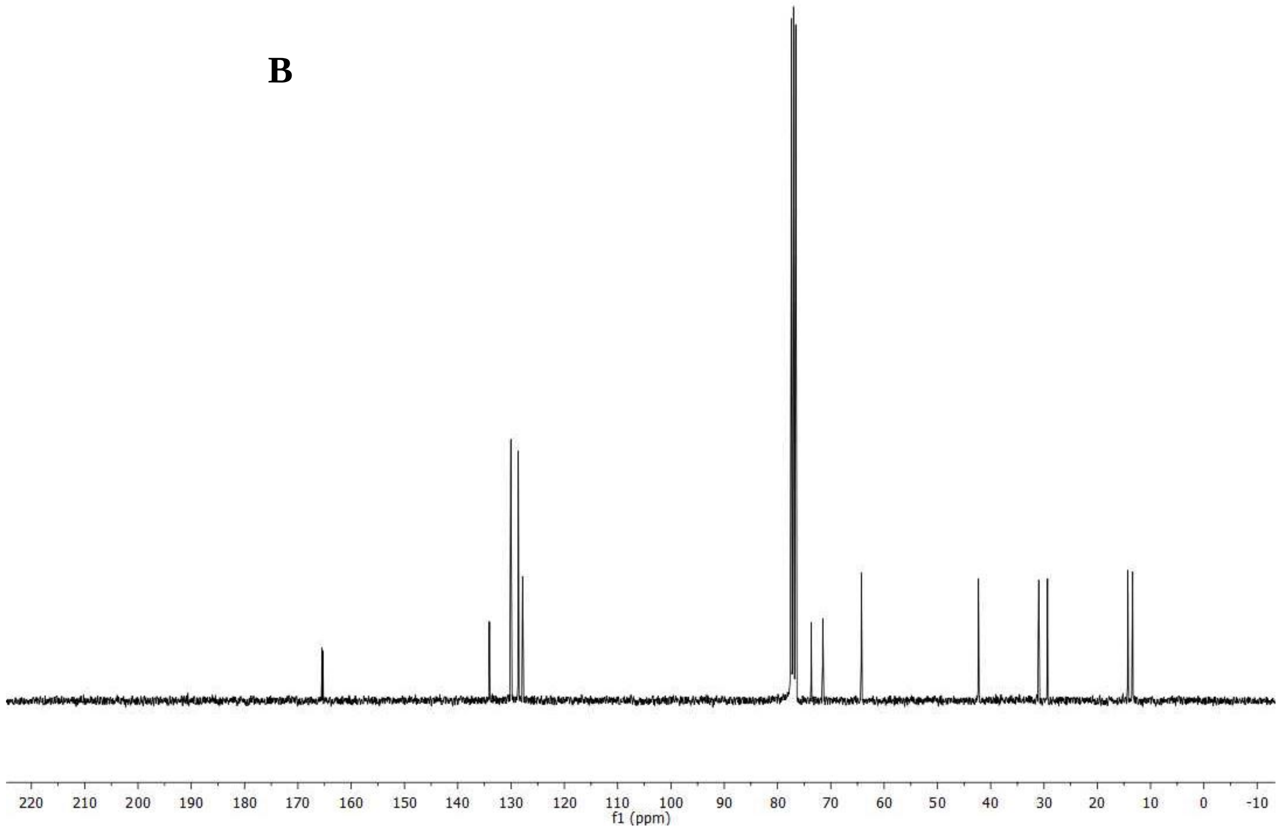

Figure S10. (**A**) 300 MHz ^{1}H NMR spectrum of **5** in $CDCl_3$; (**B**) 75 MHz ^{13}C NMR spectrum of **5** in $CDCl_3$.

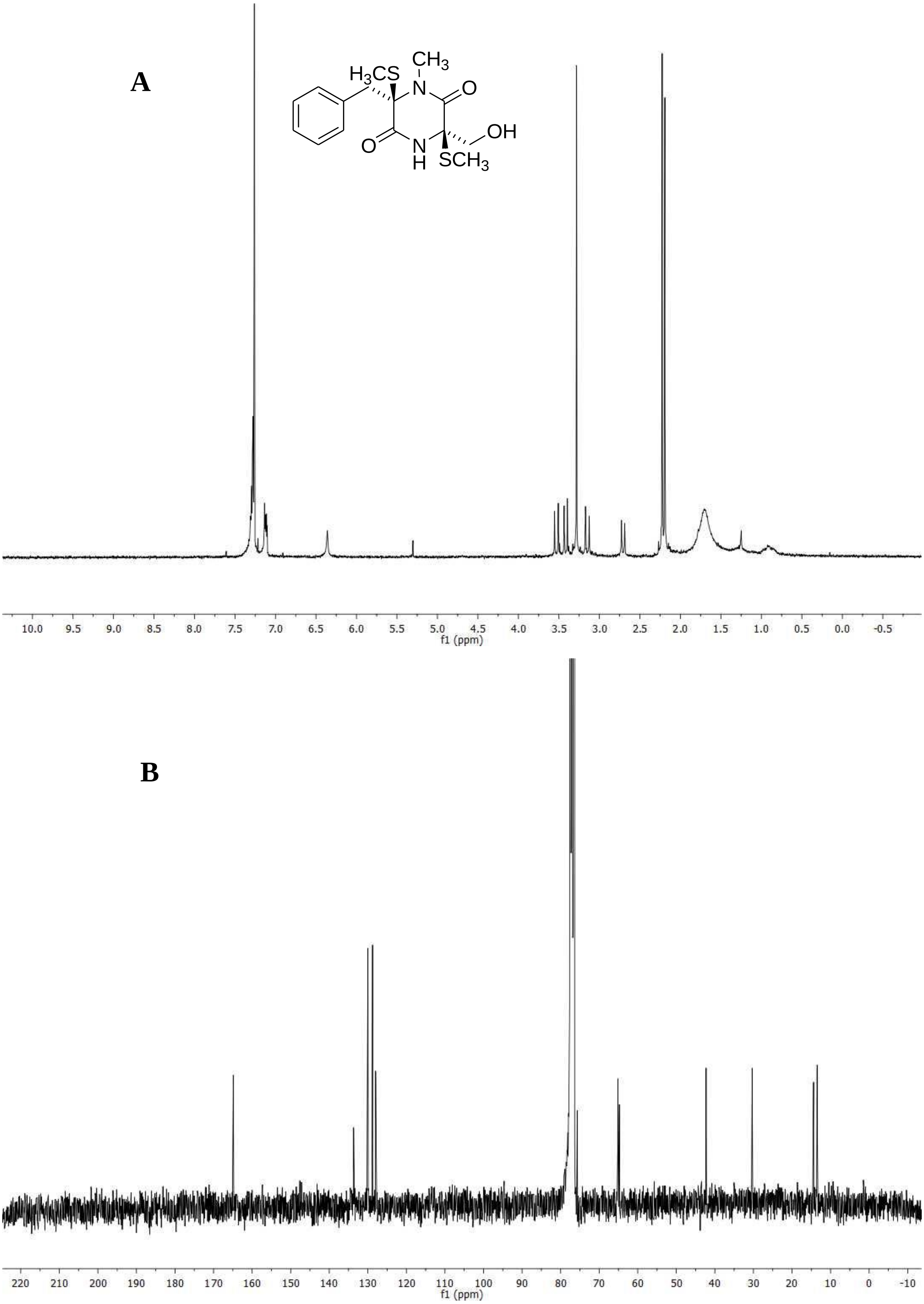

Figure S11. (**A**) 300 MHz ^{1}H NMR spectrum of **6** in d_4-MeOH; (**B**) 75 MHz ^{13}C NMR spectrum of **6** in d_4-MeOH.

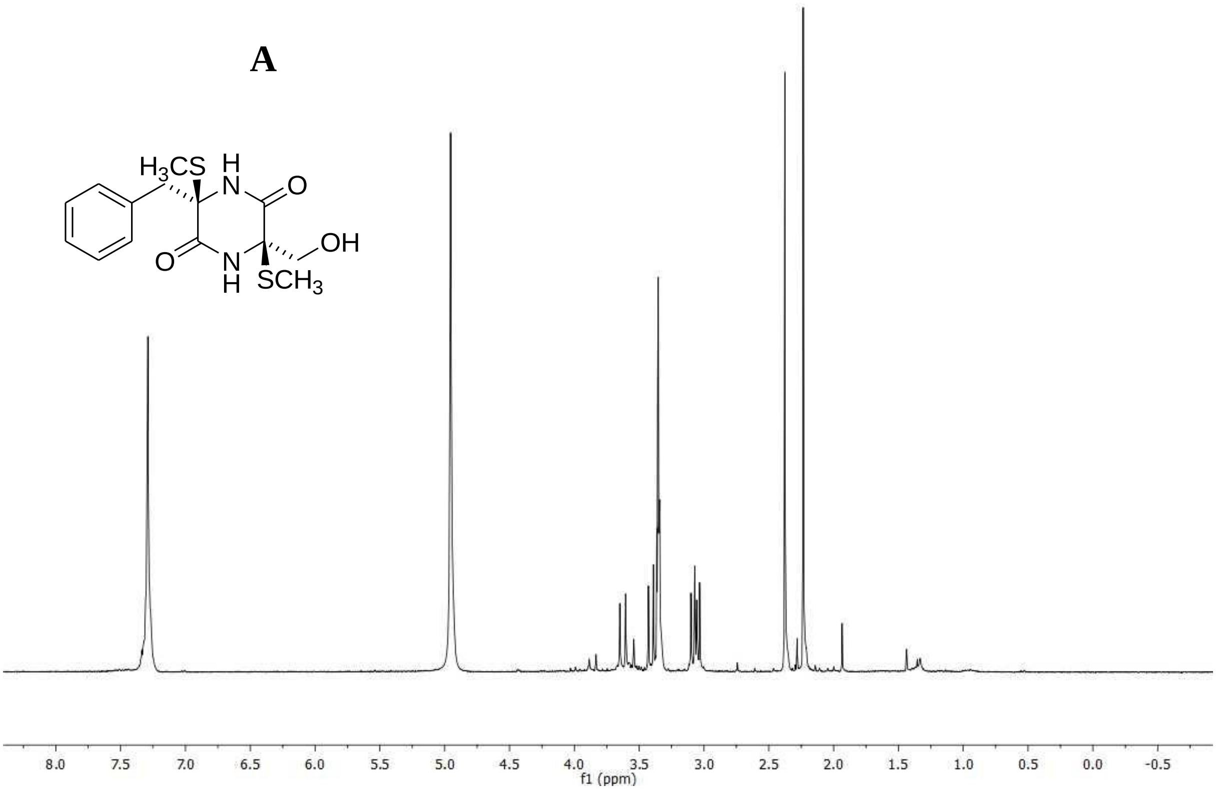

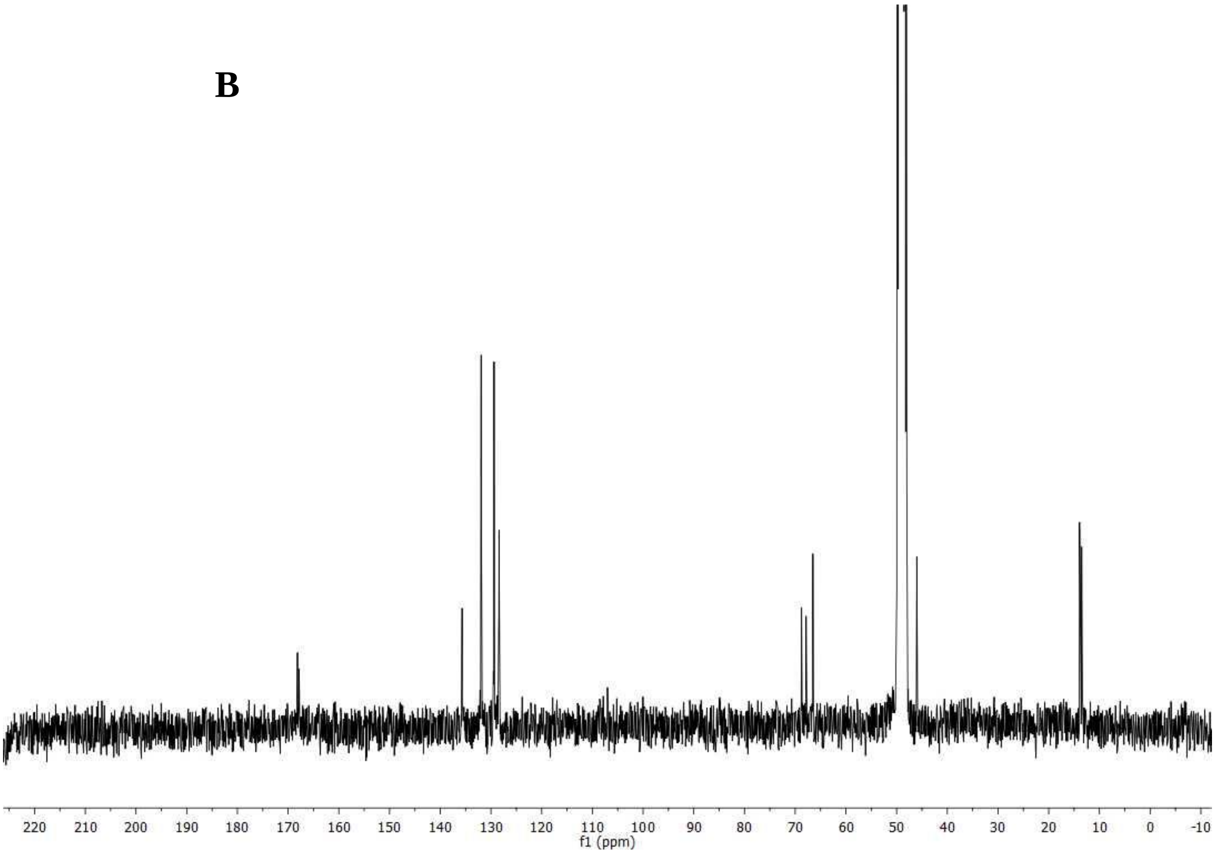

Supplement: Supplementary File 1 [file marinedrugs-10-02912-s001.pdf]
